# Statin Discontinuation and Cardiovascular Events Among Older People in Denmark

**DOI:** 10.1001/jamanetworkopen.2021.36802

**Published:** 2021-12-02

**Authors:** Wade Thompson, Lucas Morin, Dorte Ejg Jarbøl, Jacob Harbo Andersen, Martin Thomsen Ernst, Jesper Bo Nielsen, Peter Haastrup, Morten Schmidt, Anton Pottegård

**Affiliations:** 1 Research Unit of General Practice, Department of Public Health, University of Southern Denmark, Odense, Denmark; 2 Hospital Pharmacy Fyn, Odense University Hospital, Odense, Denmark; 3 Inserm CIC 1431, Clinical Investigation Unit, University Hospital of Besançon, Besançon, France; 4 Inserm 1018, High-Dimensional Biostatistics for Drug Safety and Genomics, Centre de Recherche en Épidémiologie et Santé des Populations, Villejuif, France; 5 Research Unit of Clinical Pharmacology and Pharmacy, Department of Public Health, University of Southern Denmark, Odense, Denmark; 6 Department of Clinical Epidemiology, Aarhus University Hospital, Aarhus, Denmark; 7 Department of Cardiology, Regional Hospital West Jutland, Herning, Denmark

## Abstract

**Importance:**

Statin use is common in older persons. Given uncertainties in ongoing benefit, changes in health status, and shifting goals of care and preferences, statin discontinuation may be considered in some older persons, although there is currently little evidence to guide this decision.

**Objective:**

To evaluate the association between statin discontinuation and the rate of major adverse cardiovascular events (MACE) among people aged 75 years or older who receive long-term statin treatment.

**Design, Setting, and Participants:**

This cohort study included all persons in Denmark aged 75 years or older who were treated with statins for at least 5 consecutive years as of January 1, 2011. Participants were followed up until December 31, 2016. Data were analyzed from July to November, 2020.

**Exposure:**

Statin discontinuation.

**Main Outcomes and Measures:**

Rate of occurrence of MACE and its components (myocardial infarction, ischemic stroke or transient ischemic attack, coronary revascularization, and death due to myocardial infarction or ischemic stroke) in persons continuing statins compared with those discontinuing statins. Confounding adjustment was done using inverse probability of treatment weighting. Analyses were conducted separately for primary prevention (no history of cardiovascular disease) and secondary prevention (history of cardiovascular disease).

**Results:**

The study included 67 418 long-term statin users, including 27 463 in the primary prevention analysis (median age, 79 years [IQR, 77-83 years]; 18 134 [66%] female) and 39 955 in the secondary prevention analysis (median age, 80 years [IQR, 77-84 years]; 18 717 [47%] female). In both primary and secondary prevention analyses, the rate of MACE was higher among persons who discontinued statins compared with those who continued statins. In the primary prevention cohort, the weighted rate difference was 9 per 1000 person-years (95% CI, 5-12 per 1000 person-years) and the adjusted sub–hazard ratio was 1.32 (95% CI, 1.18-1.48), corresponding to 1 excess MACE per 112 persons who discontinued statins per year. In the secondary prevention cohort, the weighted rate difference was 13 per 1000 person-years (95% CI, 8-17 per 1000 person-years) and the adjusted sub–hazard ratio was 1.28 (95% CI, 1.18-1.39), corresponding to 1 excess MACE per 77 persons who discontinued statins per year.

**Conclusions and Relevance:**

In this cohort study, among older adults receiving long-term statin treatment, discontinuation of statins was associated with a higher rate of MACE compared with statin continuation in both the primary and the secondary prevention cohorts. These findings suggest a need for robust evidence from randomized clinical trials.

## Key Points

**Question:**

Is statin discontinuation associated with a higher rate of major adverse cardiovascular events than statin continuation among older people receiving long-term treatment with statins?

**Findings:**

In this cohort study of 27 463 people treated with statins for primary prevention and 39 955 treated for secondary prevention, statin discontinuation was associated with a significantly higher rate of major adverse cardiovascular events for primary prevention and secondary prevention compared with treatment continuation.

**Meaning:**

In this study, statin discontinuation was associated with a higher rate of cardiovascular events than statin continuation among older people receiving long-term statin treatment, but more definitive evidence is needed.

## Teaser

This cohort study uses registry data from Denmark to evaluate the association between discontinuation of long-term statin treatment and major adverse cardiovascular events among individuals aged 75 years or older.

## Introduction

Statins are recommended both in persons with no history of cardiovascular disease who are at moderate or high risk of a future cardiovascular event (primary prevention) and in those with a history of cardiovascular disease, such as prior myocardial infarction (MI) or ischemic stroke (secondary prevention). The prevalence of statin use ranges from approximately 10% to 60% among persons aged 80 years or older depending on the setting.^1^ People may start taking statins in midlife and continue treatment for many years.^2^ Over this time, they may develop additional chronic diseases and their goals of care and treatment preferences may change.^3,4^ As such, guidelines suggest individualized decision-making about statin use in older persons.^5,6,7,8,9,10^ This can involve consideration of discontinuation, especially in the context of serious illness and frailty.^5,7,11,12^ Discontinuation of statins appears to be common clinical practice.^13^ Among older persons, there has been uncertainty about the benefit of statins for primary prevention and for the treatment of frail older persons and those with chronic multimorbidity,^5,7,14^ although some recent studies^15,16,17,18^ suggest possible benefit among older persons. There remains limited evidence on the potential effects of discontinuing long-term statin use. Existing studies have been conducted among persons with mean ages in the 60s or 70s^19,20,21^ and in specific contexts, particularly in palliative care^20^ and for exclusively or predominantly primary prevention.^19,21,22^ Given the lack of randomized clinical trials (RCTs) on statin discontinuation, real-world observational data can provide important evidence to support clinical decision-making.^23^ The aim of our study was to examine the association of statin discontinuation with the rate of major adverse cardiovascular events (MACE) among individuals aged 75 years or older who were prescribed long-term statins for primary and secondary prevention.

## Methods

This was a nationwide cohort study in Denmark that compared the incidence of MACE among people aged 75 years or older who discontinued statin therapy with those who continued treatment, with up to 6 years of follow-up. The study was approved by Statistics Denmark, the Danish Data Protection Agency, and the University of Southern Denmark Research and Innovation Organization. Approval from the ethics committee and informed consent was not required according to section 14 of the Act on Research Ethics Review of Health Research Projects^24^ because the study was based solely on register data. This study followed the Strengthening the Reporting of Observational Studies in Epidemiology (STROBE) reporting guideline.

### Data Sources

The data for this study were obtained from the Danish Health Data Authority. We obtained data on redeemed medications from the Danish National Prescription Registry^25^ and diagnoses from the Danish National Patient Registry, which includes primary and secondary diagnoses for a hospitalization or hospital visit (specialist, hospital-based outpatient care) and procedures.^26^ Data on cohabitation status, age, and sex were from the Danish Population Registry.^27^ Information on deaths was obtained from the Danish Register of Causes of Death.^28^

### Population

The population of interest was all Danish residents aged 75 years or older who received long-term statin treatment as of January 1, 2011, defined as persons who had filled at least 1 statin prescription annually between January 1, 2006, and December 31, 2010, and had a medication possession ratio (MPR) of 70% or greater over this period (the MPR is the ratio of the days’ supply of a medication over a particular period to the number of days in that period).^29^ Analyses were conducted separately for people treated with statins for secondary prevention (history of cardiovascular disease [January 1, 1995, to December 31, 2010 (baseline)], including MI, ischemic heart disease, angina, peripheral artery disease, ischemic stroke, and transient ischemic attack) and those taking statins for primary prevention (no history of cardiovascular disease).

### Exposures

At cohort entry (January 1, 2011), all individuals were assigned to the continuation group (continuers) and contributed time for statin continuation until either discontinuation, death, or the end of the observation period (December 31, 2016). Those who, during follow-up, did not fill another statin prescription during the number of days covered by their previous prescription plus a 180-day grace period were classified as discontinuers. The length of the grace period (180 days) was selected to minimize the risk of exposure misclassification that could arise from irregular drug filling patterns and was based on previous exploratory work.^30^ Individuals were assigned to the discontinuation group on the day after the end of the grace period and contributed time to the discontinuation group until either statin therapy reinitiation (on the day of refill), death, or December 31, 2016.

### Outcomes

The primary outcome was MACE, a composite of MI, ischemic stroke or transient ischemic attack, coronary revascularization procedure (percutaneous coronary intervention and/or coronary artery bypass graft),^31^ or death from myocardial infarction or ischemic stroke as underlying cause of death). Each of these 4 individual events were defined as secondary outcomes. Follow-up began on June 30, 2011, corresponding to January 1, 2011, plus 180 days, because people could only be classified as discontinuers after a 180-day grace period. People could only experience the first event for each type of secondary outcome, but individuals who experienced 1 type of event (other than death) were still followed up for other types of secondary outcomes. Death from any cause other than MI or ischemic stroke was treated as a competing event.^32,33^

### Statistical Analysis

Data were analyzed from July to November, 2020. We reported crude incidence rates and rate differences per 1000 person-years for all outcomes. To adjust for measured differences between statin continuers and discontinuers, we first calculated propensity scores at cohort entry (January 1, 2011) based on age, sex, cohabitation status, statin intensity, number of medications, chronic comorbidities, and concomitant medications (eTables 1-4 and eFigure 1 in the Supplement). Propensity score distribution curves were used to evaluate the degree of conditional exchangeability (eFigures 2 and 3 in the Supplement).^34^ We then used inverse probability treatment weighting with stabilized weights to create a pseudo-population in which statin discontinuation was independent of measured covariates.^35^ Covariate balance in the weighted cohorts was evaluated using standardized mean differences (eTables 5 and 6 in the Supplement).^36^ We used competing-risk regression models (Fine-Gray subdistribution hazard models) to evaluate outcomes, which incorporated the competing risk of death from causes other than MI or stroke. The relative difference in occurrence of outcomes between groups was thereafter estimated as propensity score–weighted sub–hazard ratios (HRs) with 95% CIs.^37^ Analyses were performed using Stata, version 16 (StataCorp LLC).

We conducted prespecified sensitivity analyses (eTables 7-12 in the Supplement) by varying the definition of prevalent use (increasing the MPR requirement to ≥80% and ≥90% and removing the MPR requirement). We also shortened the grace period used to define discontinuation (from 180 to 30 and 90 days). Furthermore, we adjusted for confounding using high-dimensional propensity scores based on age, sex, statin intensity, cohabitation status, and the 500 most common diagnoses and drugs.^38,39^ Finally, we calculated the E-value^40^ for MACE in the main analysis, to identify the influence of unmeasured confounders in our analysis. The E-value is the minimum strength of association that an unmeasured confounder would need to have with both the treatment (statin discontinuation) and the outcome to fully explain away the observed association between the treatment and outcome after adjustment for the measured covariates.^40^ In nonprespecified post hoc analyses (eTables 13-20 in the Supplement), we analyzed percutaneous coronary intervention and coronary artery bypass graft separately. We also compared the incidence of general practitioner visits and incidence of mortality from causes other than MI or stroke in the discontinuation and continuation groups, described use of other cardiovascular medications leading to statin discontinuation, and calculated the annual incidence of MACE. We conducted nonprespecified post hoc analyses for all-cause mortality (using a Cox proportional hazards regression model), noncardiovascular mortality, and a negative control outcome (hip fracture) using the same parameters as in the main analysis. Finally, we stratified analyses by sex.

## Results

### Characteristics of the Cohorts

We identified a total of 67 418 long-term statin users as of January 1, 2011. This included 27 463 (41%) persons without a history of cardiovascular disease (primary prevention) and 39 955 (59%) with a history of cardiovascular disease (secondary prevention). In the primary prevention cohort, the median age was 79 years (IQR, 77-83 years), and 18 134 persons (66%) were female. In the secondary prevention cohort, the median age was 80 years (IQR, 77-84 years), and 18 717 persons (47%) were female. The median duration of follow-up in the main analysis was 5.5 years (IQR, 2.8-5.5 years) in the primary prevention cohort and 4.2 years (IQR, 1.8-5.5 years) in the secondary prevention cohort. The discontinuation rate over the follow-up period the primary prevention cohort was 30% (89 311 of 279 463 persons) and in the secondary prevention cohort was 25% (9 853 of 39 955 persons). In the primary prevention group, 3085 discontinuers (37%) were censored for restarting statins, and in the secondary prevention group, 3541 (36%) were censored for restarting. Table 1 and Table 2 show full details of the study population, including comorbidities and coprescriptions.

**Table 1.** Baseline Characteristics of the Primary Prevention Cohort

Characteristic	Individuals, No. (%)				
	Overall (N = 27 463)	Before weighting		After weighting	
		Discontinuation group (n = 8311)	Continuation group (n = 19 152)	Discontinuation group (n = 8310)	Continuation group (n = 19 153)
Sex					
Female	18 134 (66.0)	5854 (70.4)	12 280 (64.1)	5487 (66.0)	12 648 (66.0)
Male	9329 (34.0)	2457 (29.6)	6872 (35.9)	2823 (34.0)	6506 (34.0)
Age, y					
Median (IQR)	79 (77-83)	80 (77-83)	79 (76-82)	79 (77-83)	79 (77-83)
75-84	23 233 (84.6)	6704 (80.7)	16 529 (86.3)	7049 (84.8)	16 169 (84.4)
≥85	4230 (15.4)	1607 (19.3)	2623 (13.7)	1261 (15.2)	2984 (15.6)
Statin intensity					
Low or moderate	26 408 (96.2)	8027 (96.6)	18 381 (96.0)	7995 (96.2)	18 420 (96.2)
High	1055 (3.8)	284 (3.4)	771 (4.0)	315 (3.8)	734 (3.8)
Unique medications					
Median (IQR)	6 (4-9)	6 (4-8)	6 (4-9)	6 (4-9)	6 (4-9)
0-4	8885 (32.4)	2866 (34.5)	6019 (31.4)	2691 (32.4)	6172 (32.2)
5-9	13 366 (48.7)	3935 (47.3)	9431 (49.2)	4006 (48.2)	9352 (48.8)
≥10	5212 (19.0)	1510 (18.2)	3702 (19.3)	1613 (19.4)	3629 (18.9)
Comorbidities					
Dementia	2858 (10.4)	1098 (13.2)	1760 (9.2)	870 (10.5)	1999 (10.4)
Diabetes	9524 (34.7)	2539 (30.5)	6985 (36.5)	2885 (34.7)	6643 (34.7)
Atrial fibrillation or flutter	2819 (10.3)	777 (9.3)	2042 (10.7)	853 (10.3)	1967 (10.3)
Heart failure	1186 (4.3)	337 (4.1)	849 (4.4)	361 (4.3)	829 (4.3)
Hypertension	9347 (34.0)	2752 (33.1)	6595 (34.4)	2845 (34.2)	6528 (34.1)
Parkinson disease	196 (0.7)	75 (0.9)	121 (0.6)	60 (0.7)	137 (0.7)
COPD	5691 (20.7)	1730 (20.8)	3961 (20.7)	1722 (20.7)	3970 (20.7)
Depression	565 (2.1)	189 (2.3)	376 (2.0)	174 (2.1)	396 (2.1)
Schizophrenia	NR	NR	NR	NR	NR
Cancer	4687 (17.1)	1399 (16.8)	3288 (17.2)	1420 (17.1)	3269 (17.1)
Medications					
Low-dose aspirin	12 480 (45.4)	3549 (42.7)	8931 (46.6)	3785 (45.5)	8708 (45.5)
ADP receptor inhibitors	297 (1.1)	93 (1.1)	204 (1.1)	90 (1.1)	208 (1.1)
Anticoagulants	2304 (8.4)	583 (7.0)	1721 (9.0)	695 (8.4)	1608 (8.4)
Thiazides	7796 (28.4)	2314 (27.8)	5482 (28.6)	2360 (28.4)	5437 (28.4)
Spironolactone	901 (3.3)	256 (3.1)	645 (3.4)	270 (3.2)	627 (3.3)
β blockers	7432 (27.1)	2063 (24.8)	5369 (28.0)	2244 (27.0)	5180 (27.0)
ACEIs or ARBs	16 297 (59.3)	4651 (56.0)	11 646 (60.8)	4936 (59.4)	11 368 (59.4)
CCBs	10 122 (36.9)	2849 (34.3)	7273 (38.0)	3070 (36.9)	7064 (36.9)
Antidepressants	4374 (15.9)	1369 (16.5)	3005 (15.7)	1331 (16.0)	3058 (16.0)
Antipsychotics	678 (2.5)	194 (2.3)	484 (2.5)	207 (2.5)	474 (2.5)
NSAIDs (nonselective)	3789 (13.8)	1163 (14.0)	2626 (13.7)	1148 (13.8)	2642 (13.8)
COX-2 inhibitors	38 (0.1)	10 (0.1)	28 (0.1)	11 (0.1)	26 (0.1)
Cohabitation status					
Alone	12 559 (45.7)	3603 (43.4)	8956 (46.8)	3794 (45.7)	8753 (45.7)
Cohabiting	14 904 (54.3)	4708 (56.6)	10 196 (53.2)	4516 (54.3)	10 400 (54.3)

Abbreviations: ACEIs, angiotensin converting enzyme inhibitors; ADP, adenosine diphosphate receptor; ARBs, angiotensin receptor blockers; CCB, calcium channel blocker; COPD, chronic obstructive pulmonary disease; COX-2, cyclooxygenase-2; NR, not reported (numbers were <10); NSAIDs, non-steroidal anti-inflammatory drugs.

**Table 2.** Baseline Characteristics of the Secondary Prevention Cohort

Characteristic	Individuals, No. (%)				
	Overall (N = 39 955)	Before weighting		After weighting	
		Discontinuation group (n = 9853)	Continuation group (n = 30 102)	Discontinuation group (n = 9855)	Continuation group (n = 30 102)
Sex					
Female	18 717 (46.8)	5261 (53.4)	13 456 (44.7)	4606 (46.7)	14 098 (46.8)
Male	21 238 (53.2)	4592 (46.6)	16 646 (55.3)	5249 (53.3)	16 005 (53.2)
Age, y					
Median (IQR)	80 (77-84)	81 (78-85)	80 (77-83)	80 (77-84)	80 (77-84)
75-84	31 931 (79.9)	7372 (74.8)	24 559 (81.6)	7837 (79.5)	24 109 (80.1)
≥85	8024 (20.1)	2481 (25.2)	5543 (18.4)	2018 (20.5)	5993 (19.9)
Statin intensity					
Low or moderate	37 469 (93.8)	9376 (95.2)	28 093 (93.3)	9239 (93.7)	28 229 (93.8)
High	2486 (6.2)	477 (4.8)	2009 (6.7)	616 (6.3)	1873 (6.2)
Unique medications					
Median (IQR)	8 (5-11)	8 (5-11)	8 (5-11)	8 (5-11)	8 (5-11)
0-4	6905 (17.3)	1855 (18.8)	5050 (16.8)	1732 (17.6)	5162 (17.1)
5-9	19 605 (49.1)	4795 (48.7)	14 810 (49.2)	4770 (48.4)	14 845 (49.3)
≥10	13 445 (33.7)	3203 (32.5)	10 242 (34.0)	3354 (34.0)	10 095 (33.5)
Comorbidities					
Dementia	4108 (10.3)	1313 (13.3)	2795 (9.3)	1022 (10.4)	3102 (10.3)
Diabetes	11 702 (29.3)	2575 (26.1)	9127 (30.3)	2900 (29.4)	8822 (29.3)
Atrial fibrillation or flutter	9079 (22.7)	2077 (21.1)	7002 (23.3)	2267 (23.0)	6849 (22.8)
Heart failure	8373 (21.0)	1873 (19.0)	6500 (21.6)	2076 (21.1)	6312 (21.0)
Hypertension	21 903 (54.8)	5344 (54.2)	16 559 (55.0)	5401 (54.8)	16 500 (54.8)
Parkinson	412 (1.0)	105 (1.1)	307 (1.0)	104 (1.1)	311 (1.0)
COPD	11 286 (28.2)	2708 (27.5)	8578 (28.5)	2800 (28.4)	8509 (28.3)
Depression	1551 (3.9)	405 (4.1)	1146 (3.8)	384 (3.9)	1170 (3.9)
Schizophrenia	16 (0.0)	NR	NR	NR	12 (0.0)
Cancer	7254 (18.2)	1738 (17.6)	5516 (18.3)	1783 (18.1)	5465 (18.2)
Medications					
Low dose aspirin	30 562 (76.5)	7294 (74.0)	23 268 (77.3)	7532 (76.4)	23 021 (76.5)
ADP receptor inhibitors	3263 (8.2)	700 (7.1)	2563 (8.5)	812 (8.2)	2460 (8.2)
Anticoagulants	5745 (14.4)	1241 (12.6)	4504 (15.0)	1436 (14.6)	4332 (14.4)
Thiazides	9160 (22.9)	2279 (23.1)	6881 (22.9)	2247 (22.8)	6897 (22.9)
Spironolactone	2687 (6.7)	553 (5.6)	2134 (7.1)	658 (6.7)	2024 (6.7)
β blockers	20 849 (52.2)	4684 (47.5)	16 165 (53.7)	5128 (52.0)	15 701 (52.2)
ACEIs or ARBs	23 018 (57.6)	5471 (55.5)	17 547 (58.3)	5687 (57.7)	17 341 (57.6)
CCBs	14 505 (36.3)	3454 (35.1)	11 051 (36.7)	3572 (36.2)	10 930 (36.3)
Antidepressants	7958 (19.9)	2001 (20.3)	5957 (19.8)	1954 (19.8)	5992 (19.9)
Antipsychotics	1007 (2.5)	251 (2.5)	756 (2.5)	254 (2.6)	761 (2.5)
NSAIDs (nonselective)	4697 (11.8)	1220 (12.4)	3477 (11.6)	1154 (11.7)	3538 (11.8)
COX-2 inhibitors	50 (0.1)	12 (0.1)	38 (0.1)	12 (0.1)	38 (0.1)
Cohabitation status					
Alone	19 571 (49.0)	4483 (45.5)	15 088 (50.1)	4836 (49.1)	14 748 (49.0)
Cohabitating	20 384 (51.0)	5370 (54.5)	15 014 (49.9)	5019 (50.9)	15 355 (51.0)

Abbreviations: ACEIs, angiotensin converting enzyme inhibitors; ADP, adenosine diphosphate receptor; ARBs, angiotensin receptor blockers; COPD, chronic obstructive pulmonary disease; CCB, calcium channel blocker; COX-2, cyclooxygenase-2; NR, not reported (numbers were <10); NSAIDs, non-steroidal anti-inflammatory drugs.

#### Primary Prevention Cohort

**Figure 1.**
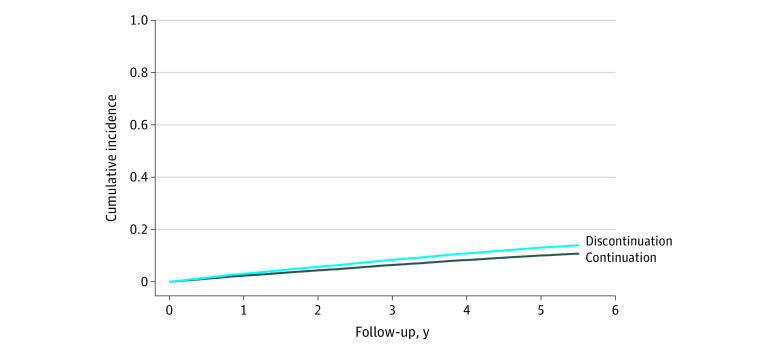
Cumulative Incidence Curve for the Outcome of Major Adverse Cardiovascular Events in the Primary Prevention Cohort

In the primary prevention cohort, the crude incidence rate of MACE was 33 per 1000 person-years (95% CI, 30-36 per 1000 person-years) in the discontinuation group and 24 per 1000 person-years (95% CI, 23-25 per 1000 person-years) in the continuation group (Table 3 and Figure 1). The crude difference in the rate of MACE was 9 per 1000 person-years (95% CI, 5-12 per 1000 person-years), and the weighted rate difference was 9 per 1000 person-years (95% CI, 5-12 per 1000 person-years), corresponding to 1 excess MACE per 112 discontinuers per year. The rate of occurrence of MACE was higher in the discontinuation group than in the continuation group (HR, 1.32; 95% CI 1.18-1.48). The rates of occurrence of MI (HR, 1.37; 95% CI, 1.11-1.70), ischemic stroke or transient ischemic attack (HR, 1.33; 95% CI, 1.14-1.54), and death due to MI or stroke (HR, 1.43; 95% CI, 1.11-1.85) were higher in the discontinuation group than in the continuation group, and there was no significant difference in the rate of occurrence of revascularization (HR, 1.12; 95% CI, 0.82-1.52) (Table 3).

**Table 3.** Summary of Results for the Primary and Secondary Prevention Cohorts

Outcome	Discontinuation group		Continuation group		Crude rate difference, per 1000 person-years (95% CI)	Weighted rate difference, per 1000 person-years (95% CI)^a^	Sub–hazard ratio (95% CI)^a^
	Crude events	Crude incidence rate, per 1000 person-years (95% CI)	Crude events	Crude incidence rate, per 1000 person-years (95% CI)			
**Primary prevention**							
Person-years of follow-up^b^	11 709	NA	103 664	NA	NA	NA	NA
MACE	382	33 (30 to 36)	2481	24 (23 to 25)	9 (5 to 12)	9 (5 to 12)	1.32 (1.18 to 1.48)
MI	105	9 (7 to 11)	692	6 (6 to 7)	2 (0 to 4)	2 (1 to 4)	1.37 (1.11 to 1.70)
Stroke	230	19 (17 to 22)	1390	13 (13 to 14)	6 (4 to 9)	6 (3 to 8)	1.33 (1.14 to 1.54)
Revascularization procedure	47	4 (3 to 5)	477	4 (4 to 5)	−1 (−2 to 1)	0 (−1 to 2)	1.12 (0.82 to 1.52)
Death from MI or stroke	76	6 (5 to 8)	433	4 (4 to 4)	2 (1 to 4)	2 (1 to 4)	1.43 (1.11 to 1.85)
**Secondary prevention**							
Person-years of follow-up^b^	12 350	NA	133 374	NA	NA	NA	NA
MACE	739	60 (56 to 64)	6472	49 (47 to 50)	11 (7 to 16)	13 (8 to 17)	1.28 (1.18 to 1.39)
MI	248	19 (17 to 21)	2326	17 (16 to 17)	2 (−0 to 5)	4 (1 to 6)	1.25 (1.09 to 1.43)
Stroke	382	30 (27 to 33)	2953	21 (21 to 22)	8 (5 to 12)	8 (5 to 11)	1.34 (1.20 to 1.50)
Revascularization procedure	74	6 (4 to 7)	1416	10 (10 to 11)	−5 (−6 to −3)	−3 (−5 to −2)	0.73 (0.57 to 0.93)
Death from MI or stroke	211	16 (14 to 18)	1387	10 (9 to 10)	6 (4 to 8)	6 (4 to 8)	1.57 (1.35 to 1.83)

Abbreviations: MACE, major adverse cardiovascular events; MI, myocardial infarction; NA, not applicable.

^a^ Adjusted estimates were obtained using an inverse probability of treatment weighted pseudo-population with stabilized weights. Weights incorporated propensity scores calculated from baseline age, sex, number of concomitant medications, individual medications, comorbidities, statin intensity, and cohabitation status.

^b^ Person-years of follow-up for MACE outcome.

#### Secondary Prevention Cohort

**Figure 2.**
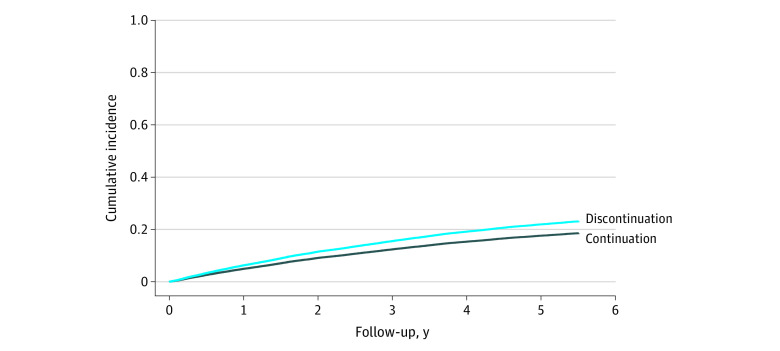
Cumulative Incidence Curve for the Outcome of Major Adverse Cardiovascular Events in the Secondary Prevention Cohort

In the secondary prevention cohort, the crude incidence rate of MACE was 60 per 1000 person-years (95% CI, 56-64 per 1000 person-years) in the discontinuation group and 49 per 1000 person-years (95% CI, 47-50 per 1000 person-years) in the continuation group (Table 3 and Figure 2). The crude difference in the rate of MACE was 11 per 1000 person-years (95% CI, 7-16 per 1000 person-years), and the weighted rate difference was 13 per 1000 person-years (95% CI, 8-17 per 1000 person-years), corresponding to 1 excess MACE per 77 discontinuers per year. The rate of occurrence of MACE was higher in the discontinuation group than in the continuation group (HR, 1.28; 95% CI, 1.18-1.39). The rates of occurrence of MI (HR, 1.25; 95% CI, 1.09-1.43), ischemic stroke or transient ischemic attack (HR, 1.34; 95% CI, 1.20-1.50), and death due to MI or stroke (HR, 1.57; 95% CI, 1.35-1.83) were higher in the discontinuation group than in the continuation group (Table 3). The rate of occurrence of revascularization was lower in the discontinuation group (HR, 0.73; 95% CI, 0.57-0.93), which was attributable to a lower rate of coronary artery bypass graft (eTable 13 in the Supplement).

### Supplementary Analyses

The results of all supplementary and post hoc analyses are given in eTables 7 to 20 in the Supplement. In the primary prevention cohort, modifying the grace period for discontinuation, changing the MPR requirement to 80% or removing it completely, or using high-dimensional propensity score had little effect on the results (eTables 7-12 in the Supplement). However, increasing the MPR requirement to 90% before cohort entry lowered the adjusted HR for MACE to 1.21 (95% CI, 1.04-1.39). Decreasing the grace period to 30 days led to a nonsignificant decrease in HR for revascularizations (0.77; 95% CI, 0.53-1.11) but a higher HR for death due to MI or stroke (HR, 1.98; 95% CI, 1.44-2.72). The E-value for MACE in the main analysis was 1.97, indicating that an unmeasured confounder would need to be associated with discontinuation and MACE by approximately 2-fold beyond the measured confounders to fully explain away our results.

In the secondary prevention cohort, post hoc analyses showed a lower rate of coronary artery bypass graft but a higher rate of percutaneous coronary intervention in the discontinuation group compared with the continuation group; however, neither difference was statistically significant (eTable 13 in the Supplement). Modifying the MPR requirement or using a high-dimensional propensity score did not substantially alter our results (eTables 7-12 in the Supplement). The E-value for MACE in the main analysis was 1.88.

In our post hoc analyses, statin discontinuation was associated with a higher rate of all-cause mortality and noncardiovascular mortality (eTable 18 in the Supplement). In our negative control outcome analysis (eTable 19 in the Supplement), statin discontinuation was associated with a higher rate of hip fracture in both the primary and the secondary prevention cohorts. In the sex-stratified analyses (eTable 20 in the Supplement), discontinuation of statin therapy prescribed for primary prevention was associated with a slightly higher absolute rate of MACE in men compared with women, whereas the rates in the secondary prevention cohort were similar between men and women.

## Discussion

In this cohort study, statin discontinuation was associated with a higher rate of occurrence of MACE compared with statin continuation among older people receiving long-term statin treatment for both primary and secondary prevention. Although the relative effect estimates for statin discontinuation were similar in both cohorts, the overall rate of cardiovascular events and the magnitude of the rate difference between discontinuation and continuation were higher in the secondary prevention cohort than among statin users without a history of cardiovascular disease.

### Comparison to Existing Literature

Few studies have explored the effects of statin discontinuation in older persons. One RCT demonstrated that, among patients with advanced illness and a physician-estimated life expectancy of less than a year, statin discontinuation did not increase risk of mortality over 60 days.^20^ Although useful, that study did not inform clinical decision-making for older adults who are, in general, not nearing the end of life.

In a recent observational study conducted in France^19^ that included older persons aged 75 years with no history of cardiovascular disease, statin discontinuation was associated with an increased risk of hospitalizations for cardiovascular events during the 2.5-year follow-up (HR, 1.33; 95% CI, 1.18-1.50). Rea et al^22^ also reported that statin discontinuation was associated with an increased risk of cardiovascular events (HR, 1.14; 95% CI, 1.03-1.26) in a predominantly primary prevention population. Our findings about the association between statin discontinuation and the rate of MACE cannot be directly compared with evidence on statin initiation during older age; our study included long-term statin users (who are not fully comparable to treatment-naive patients), and the time to benefit associated with initiating therapy may be substantially longer than the time to harm associated with withdrawing that same medication. Furthermore, long-term statin use may have a substantial treatment benefit that persists after a trial has ended (ie, legacy benefit), as suggested by a recent meta-analysis of RCTs.^41^

Survey studies and qualitative literature suggest that patient preferences, physical frailty, and remaining life expectancy can influence prescribers’ decisions about statin discontinuation.^42,43^ Therefore, confounding by indication and prognosis bias are 2 important concerns in drug discontinuation studies relying on observational data. For instance, statin discontinuation may reflect an overall preference for less medical care and a willingness to shift from preventive or curative goals of care to a more palliative approach. Poor health and severe frailty may also be associated with an increased likelihood of statin discontinuation^43,44^ and with a reduced likelihood of receiving revascularization procedures, particularly coronary artery bypass graft.^45,46,47^ Our post hoc analyses support this hypothesis because we found a lower rate of coronary artery bypass graft in the discontinuation group than in the continuation group, especially in the secondary prevention cohort. The finding that statin discontinuation was associated with a higher risk of hip fracture (negative control analysis) and with higher rates of all-cause and noncardiovascular mortality also support the hypothesis that statin discontinuation is a marker of frailty and poorer health in general.

### Implications and Future Research

Our results provide important evidence on statin discontinuation in people receiving long-term statin treatment for both primary and secondary prevention. Clinicians and policy makers should be aware of a possible increased risk of MACE associated with discontinuation of long-term statin treatment. This finding may be of clinical and public health importance because of the magnitude of excess risk (1 excess MACE per 112 discontinuers per year in the primary prevention cohort and 1 MACE per 77 discontinuers per year in the secondary prevention cohort). Consideration of absolute risk is particularly important for shared decision-making in the context of statin use for individual patients.

Statin discontinuation occurs frequently in practice,^1^ and thus evidence to inform shared decision-making on this topic may be useful to clinicians. Our findings of a higher rate of cardiovascular events among those discontinuing statins, along with the findings of previous studies,^19,22^ highlight the importance of RCTs on this topic. To our knowledge, there is currently only 1 RCT^23^ that has investigated the effects of statin discontinuation for primary prevention among persons aged 75 years or older. Additional RCTs investigating statin initiation in primary prevention for older persons are also ongoing and should provide additional context about statins in this population.^48,49^

### Limitations

This study has limitations. First, no data were available about the specific reasons that prompted statin discontinuation. The discontinuation patterns that we captured in the data may represent either a purposeful and planned deprescribing process or patients stopping therapy on their own without consulting a health care professional. Second, we did not incorporate time-varying confounding into our analysis. Cardiovascular medications may have changed during the study period, or people may have developed additional comorbidities that may have influenced statin discontinuation and the risk of acute cardiovascular outcomes. Third, statin exposure was defined based on redeemed prescriptions only. Unmeasured confounding is a concern in any non-RCT. However, the sensitivity analysis using the high-dimensional propensity score (adjusting for the 500 most common medications and comorbidities) did not change the results. We could only incorporate potential confounders available to us in Danish registers and thus could not include lifestyle factors, patient preferences, and other potential confounders. Frailty may be a particularly relevant confounder. A negative control outcome analysis revealed an association of statin discontinuation with hip fracture, highlighting that discontinuation may be a marker of frailty in general. However, an E-value for 2 of the main analyses suggested that an unmeasured confounder would need to be associated with discontinuation and with MACE by 2-fold each beyond the measured confounders to explain away our results.^40^ The validity of cardiovascular diagnosis and procedure codes is another potential consideration; however, the validity of cardiovascular diagnosis^50^ and procedure codes^31^ has been demonstrated to be excellent in Danish health registers. Thus, we do not believe that this affected our results. In addition, our post hoc analyses of all-cause mortality and noncardiovascular mortality should be interpreted with caution because these outcomes are particularly prone to confounding by indication and thus were not selected as prespecified outcomes.

## Conclusions

In this cohort study, among older Danes receiving long-term statin treatment, discontinuation was associated with a higher rate of MACE compared with statin continuation. Although the relative effect of statin discontinuation was similar in the primary and secondary prevention cohorts, the rate difference was larger in the secondary prevention cohort. These findings suggest a need for further evidence from RCTs on this topic to inform shared decision-making in clinical practice.

## Notes

Supplement. **eTable 1.** Anatomical Therapeutic Chemical codes used in the study **eTable 2.** International Classification of Diseases codes used in the study **eTable 3.** Surgical procedure codes used in the study **eTable 4.** Statin intensity (based on American College of Cardiologists 2018 guidelines) **eFigure 1.** Cohort design **eFigure 2.** Propensity score distribution in the primary prevention cohort **eFigure 3.** Propensity score distribution in the secondary prevention cohort **eTable 5.** Balance of covariates in primary prevention cohort **eTable 6.** Balance of covariates in secondary prevention cohort **eTable 7.** Summary of results with grace period of 90 days **eTable 8.** Summary of results with grace period of 30 days **eTable 9.** Summary of results with medication possession ratio threshold of ≥80% **eTable 10.** Summary of results with medication possession ratio threshold of ≥90% **eTable 11.** Summary of results with no medication possession ratio threshold **eTable 12.** Summary of results using high dimensional propensity score in inverse probability of treatment weighting procedure **eTable 13.** Revascularization outcome broken down by PCI and CABG **eTable 14.** General Practitioner (GP) contacts each year of follow-up **eTable 15.** Crude incidence of mortality for causes other than MI and ischemic stroke **eTable 16.** Other cardiovascular medication use approaching statin discontinuation date in statin discontinuation group **eTable 17.** Crude incidence of MACE each year of follow-up **eTable 18.** Summary of results for all-cause and non-cardiovascular mortality **eTable 19.** Summary of results for negative control outcome (hip fracture) **eTable 20.** Summary of results sex-stratified MACE analysis
