# Data Errors in Results Section

**DOI:** 10.1001/jamanetworkopen.2022.0010

**Published:** 2022-01-28

In the Original Investigation titled “Statin Discontinuation and Cardiovascular Events Among Older People in Denmark,”^1^ published December 2, 2021, in the first paragraph of the Results section, the number of patients in the primary prevention cohort who discontinued statins should have been given as 8311 of 27 463, not 89 311 of 279 463. This article has been corrected.^1^
