# Supplementary material for: Statin Discontinuation and Cardiovascular Events Among Older People in Denmark
Source: JAMA Netw Open. 2021 Dec 2;4(12):e2136802. doi: 10.1001/jamanetworkopen.2021.36802 (PMC8640890; doi:10.1001/jamanetworkopen.2021.36802)
Supplement: Supplement. — eTable 1. Anatomical Therapeutic Chemical codes used in the study eTable 2. International Classification of Diseases codes used in the study eTable 3. Surgical procedure codes used in the study eTable 4. Statin intensity (based on American College of Cardiologists 2018 guidelines) eFigure 1. Cohort design eFigure 2. Propensity score distribution in the primary prevention cohort eFigure 3. Propensity score distribution in the secondary prevention cohort eTable 5. Balance of covariates in primary prevention cohort eTable 6. Balance of covariates in secondary prevention cohort eTable 7. Summary of results with grace period of 90 days eTable 8. Summary of results with grace period of 30 days eTable 9. Summary of results with medication possession ratio threshold of ≥80% eTable 10. Summary of results with medication possession ratio threshold of ≥90% eTable 11. Summary of results with no medication possession ratio threshold eTable 12. Summary of results using high dimensional propensity score in inverse probability of treatment weighting procedure eTable 13. Revascularization outcome broken down by PCI and CABG eTable 14. General Practitioner (GP) contacts each year of follow-up eTable 15. Crude incidence of mortality for causes other than MI and ischemic stroke eTable 16. Other cardiovascular medication use approaching statin discontinuation date in statin discontinuation group eTable 17. Crude incidence of MACE each year of follow-up eTable 18. Summary of results for all-cause and non-cardiovascular mortality eTable 19. Summary of results for negative control outcome (hip fracture) eTable 20. Summary of results sex-stratified MACE analysis [file jamanetwopen-e2136802-s001.pdf]

Supplemental Online Content

Thompson W, Morin L, Jarbøl DE, et al. Statin discontinuation and cardiovascular events among older people in Denmark. *JAMA Netw Open.* 2021;4(12):e2136802. doi:10.1001/jamanetworkopen.2021.36802

eTable 1. Anatomical Therapeutic Chemical codes used in the study

eTable 2. International Classification of Diseases codes used in the study

eTable 3. Surgical procedure codes used in the study

eTable 4. Statin intensity (based on American College of Cardiologists 2018 guidelines)

eFigure 1. Cohort design

eFigure 2. Propensity score distribution in the primary prevention cohort

eFigure 3. Propensity score distribution in the secondary prevention cohort

eTable 5. Balance of covariates in primary prevention cohort

eTable 6. Balance of covariates in secondary prevention cohort

eTable 7. Summary of results with grace period of 90 days

eTable 8. Summary of results with grace period of 30 days

eTable 9. Summary of results with medication possession ratio threshold of ≥80%

eTable 10. Summary of results with medication possession ratio threshold of ≥90%

eTable 11. Summary of results with no medication possession ratio threshold

eTable 12. Summary of results using high dimensional propensity score in inverse probability of treatment weighting procedure

eTable 13. Revascularization outcome broken down by PCI and CABG

eTable 14. General Practitioner (GP) contacts each year of follow-up

eTable 15. Crude incidence of mortality for causes other than MI and ischemic stroke

eTable 16. Other cardiovascular medication use approaching statin discontinuation date in statin discontinuation group

eTable 17. Crude incidence of MACE each year of follow-up

eTable 18. Summary of results for all-cause and non-cardiovascular mortality

eTable 19. Summary of results for negative control outcome (hip fracture)

eTable 20. Summary of results sex-stratified MACE analysis

© 2021 Thompson W et al. *JAMA Network Open.*

This supplemental material has been provided by the authors to give readers additional information about their work.

eTable 1. Anatomical Therapeutic Chemical codes used in the study.

Medication class	ATC code(s)
Statins	C10AA, C10BA02, C10BA05
Low dose ASA	B01AC06
ADP inhibitors	B01AC04, B01AC05, B01AC22, B01AC24, B01AC25
Anticoagulants (warfarin, NOACs)	B01AA03, B01AE07, B01AF01, B01AF02, B01AF03
Thiazides	C03AA, C03AB
Spironolactone	C03DA01
Beta-blockers	C07A
ACE inhibitors/ARBS	C09A, C09B, C09C, C09D
Calcium channel blockers	C08CA, C08D
Antidepressants	N06A
Antipsychotics	N05A (except N05AN)
Non-selective NSAIDs	M01A (except M01AH, M01AX05, M01AX25)
COX-2 inhibitors	M01AH
COPD medications*	R03AC12, R03AC13, R03AC1, R03AC19, R03CC12, R03BB04, R03BB05, R03BB06, R03BB07, R03BA01, R03BA02, R03BA03, R03BA05, R03BA07, R03BA08, R03AK06, R03AK07, R03AK08, R03AK10, R03AK11, R03AL03, R03AL04, R03AL05, R03AL06
Diabetes medications*	A10B, A10A
Dementia medications*	N06DA, N06DX01

* Only used for disease definitions

Abbreviations: ACE = angiotensin converting enzyme; ADP = adenosine diphosphate receptor; ARB = angiotensin receptor blocker; ASA = acetylsalicylic acid; ATC = anatomical therapeutic chemical; COPD = chronic obstructive pulmonary disease; COX-2 = cyclooxygenase-2; NOAC = new oral anticoagulant; NSAID = non-steroidal anti-inflammatory drugs; SMD = standardized mean difference

eTable 2. International Classification of Diseases codes used in the study.

Disease	ICD-10 code
MI	I21 I22 I23
Ischemic stroke/TIA	I63 G45
Ischemic heart disease/angina	I20 I25
Peripheral artery disease	I739, I702
"Secondary prevention"	Any of: MI, ischemic stroke/TIA, ischemic heart disease/ angina, peripheral artery disease
Dementia	F01, F02, F03, G30, G310, G3183, use of dementia medication (see above)
Diabetes	E10, E11, use of diabetes medication (see above)
Atrial fibrillation/flutter	I48
Hypertension	I10, I11, I12, I13, I14, I15
Parkinson's disease	G20
COPD	J41, J42, J43, J44, use of COPD medication (see above)
Depression	F32, F33
Schizophrenia	F20
Cancer	C00 to C96
Heart failure	I50, I110, I130, I132

Abbreviations: COPD = chronic obstructive pulmonary disease; ICD = international classification of diseases; MI = myocardial infarction; TIA = transient ischemic attack

eTable 3. Surgical procedure codes used in the study.

Procedure	Code
Percutaneous coronary intervention	KFNG, KFNF
Coronary artery bypass graft	KFNA-KFNE, KFNH20

© 2021 Thompson W et al. *JAMA Network Open.*

eTable 4. Statin intensity (based on American College of Cardiologists 2018 guidelines).

Statin	High intensity	Low/moderate
Atorvastatin (C10AA05)	40 to 80 mg/day	10 to 20 mg/day
Fluvastatin (C10AA04)	-	20 to 80 mg/day
Lovastatin (C10AA02)	-	20 to 40 mg/day
Pravastatin (C10AA03)	-	10 to 80 mg/day
Rosuvastatin (C10AA07)	20 to 40 mg/day	5 to 10 mg/day
Simvastatin (C10AA01)	-	10 to 40 mg/day

eFigure 1. Cohort design.

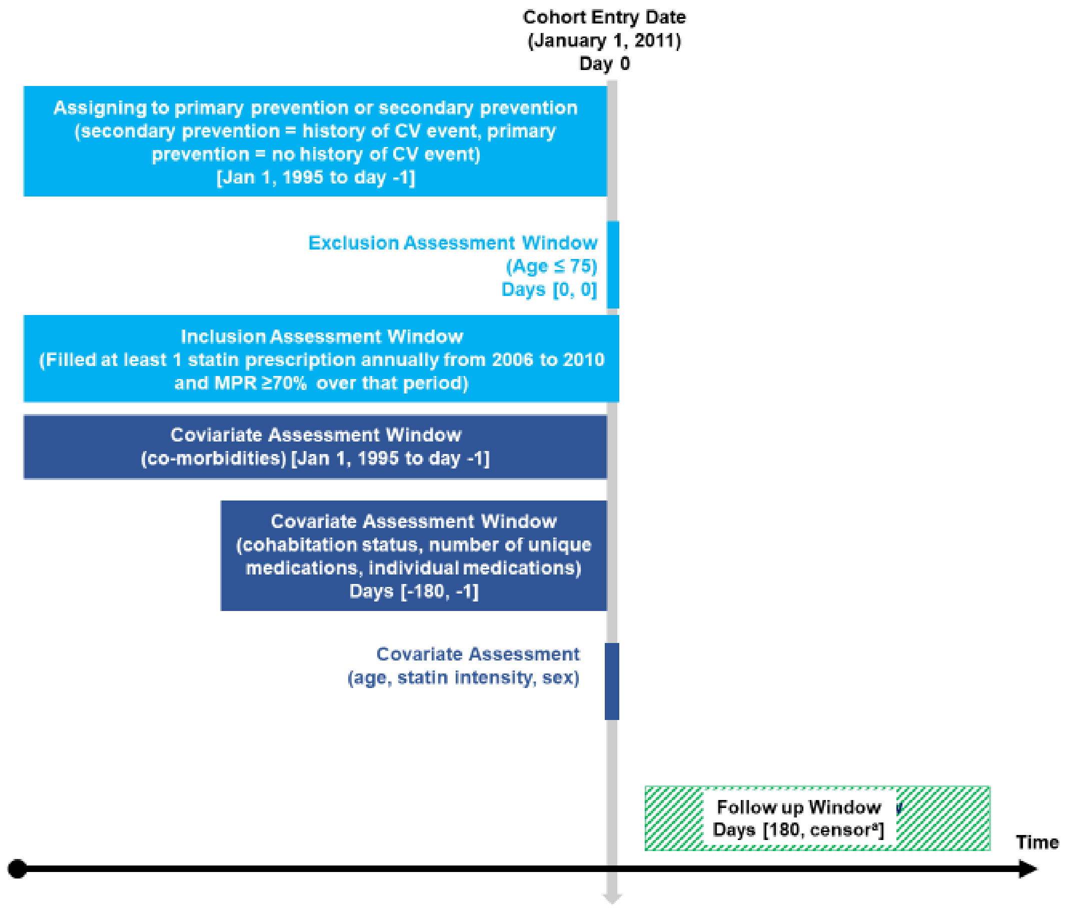

a. Censored if experienced outcome of interest, discontinued then restarted statin, competing event (death), or end of study period

© 2021 Thompson W et al. *JAMA Network Open.*

eFigure 2. Propensity Score Distribution in the Primary Prevention Cohort.

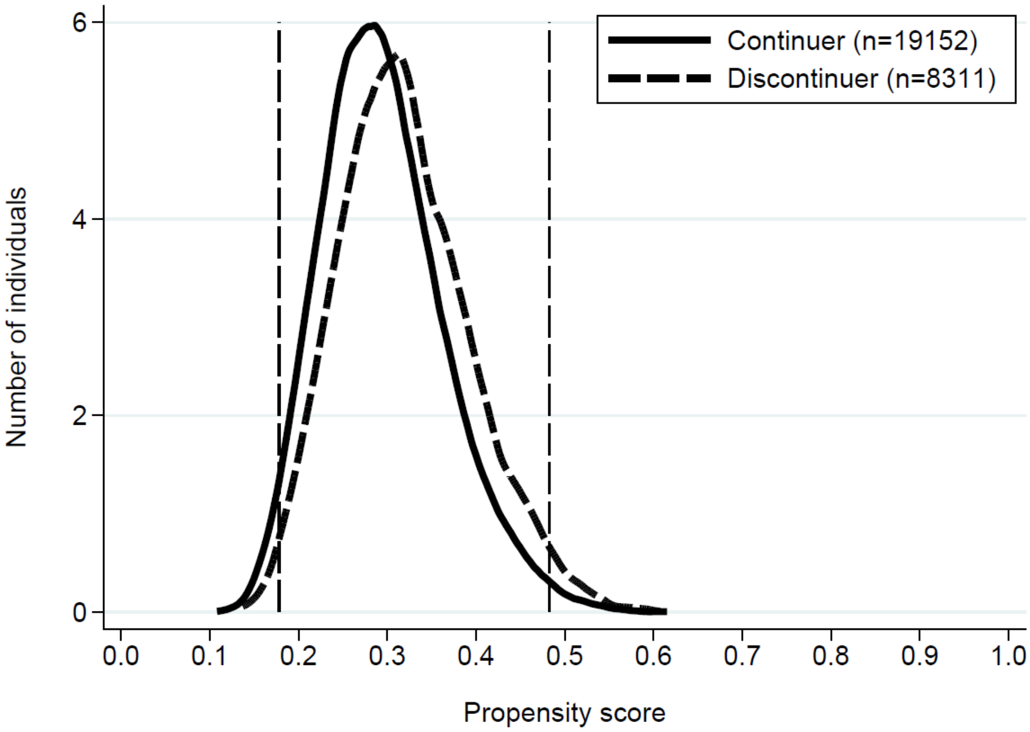

© 2021 Thompson W et al. *JAMA Network Open.*

eFigure 3. Propensity Score Distribution in the Secondary Prevention Cohort.

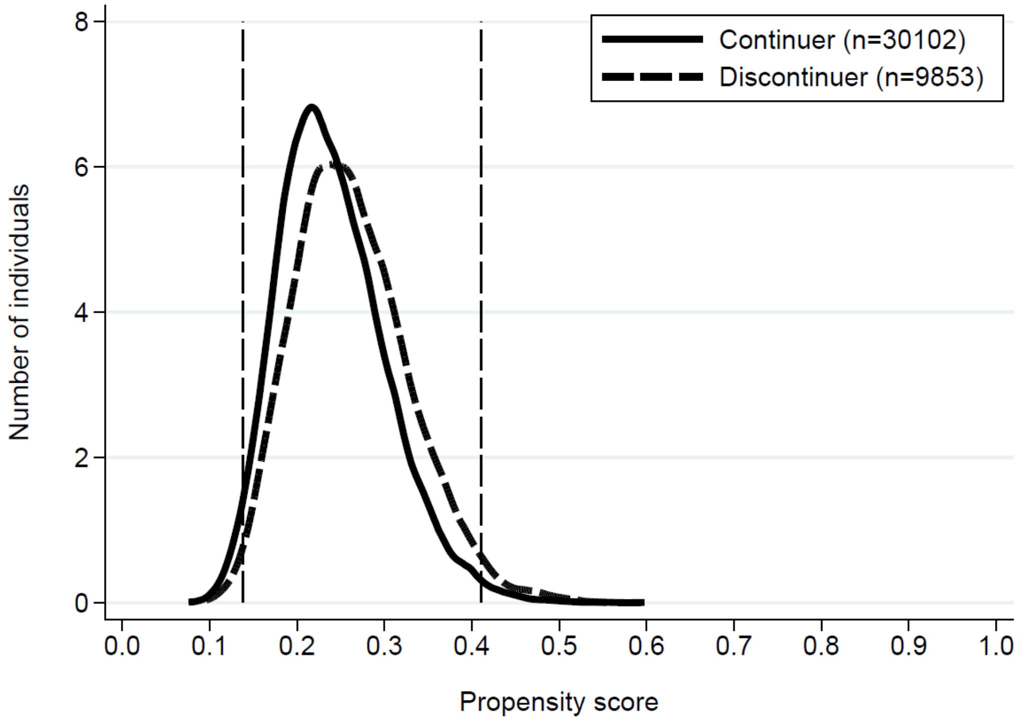

© 2021 Thompson W et al. *JAMA Network Open.*

eTable 5. Balance of Covariates in Primary Prevention Cohort.

		Before weighting			After weighting		
	**Overall**	**Discontinuation**	**Continuation**	**SMD**	**Discontinuation**	**Continuation**	**SMD**
	(n=27,463)	(n=8,311)	(n=19,152)		(n=8,310)	(n=19,153)	
**Sex (n,%)**							
Female	18,134 (66.0)	5,854 (70.4)	12,280 (64.1)	0.03	5,487 (66.0)	12,648 (66.0)	0.00
Male	9,329 (34.0)	2,457 (29.6)	6,872 (35.9)	0.01	2,823 (34.0)	6,506 (34.0)	0.00
**Age**							
Median (IQR)	79 (77-83)	80 (77-83)	79 (76-82)	0.22	79 (77-83)	79 (77-83)	0.00
75-84 y (n,%)	23,233 (84.6)	6,704 (80.7)	16,529 (86.3)	0.04	7,049 (84.8)	16,169 (84.4)	0.01
≥85 y (n,%)	4,230 (15.4)	1,607 (19.3)	2,623 (13.7)	0.04	1,261 (15.2)	2,984 (15.6)	0.01
**Statin intensity (n,%)**							
Low/moderate	26,408 (96.2)	8,027 (96.6)	18,381 (96.0)	0.07	7,995 (96.2)	18,420 (96.2)	0.00
High	1,055 (3.8)	284 (3.4)	771 (4.0)	0.06	315 (3.8)	734 (3.8)	0.00
**Unique medications**							
Median (IQR)	6 (4-9)	6 (4-8)	6 (4-9)	0.05	6 (4-9)	6 (4-9)	0.02
0-4	8,885 (32.4)	2,866 (34.5)	6,019 (31.4)	0.06	2,691 (32.4)	6,172 (32.2)	0.00
5-9	13,366 (48.7)	3,935 (47.3)	9,431 (49.2)	0.10	4,006 (48.2)	9,352 (48.8)	0.01
≥10	5,212 (19.0)	1,510 (18.2)	3,702 (19.3)	0.06	1,613 (19.4)	3,629 (18.9)	0.01
**Comorbidities, (n,%)**							
Dementia	2,858 (10.4)	1,098 (13.2)	1,760 (9.2)	0.02	870 (10.5)	1,999 (10.4)	0.00
Diabetes	9,524 (34.7)	2,539 (30.5)	6,985 (36.5)	0.06	2,885 (34.7)	6,643 (34.7)	0.00
Atrial fibrillation / flutter	2,819 (10.3)	777 (9.3)	2,042 (10.7)	0.09	853 (10.3)	1,967 (10.3)	0.00
Heart failure	1,186 (4.3)	337 (4.1)	849 (4.4)	0.13	361 (4.3)	829 (4.3)	0.00
Hypertension	9,347 (34.0)	2,752 (33.1)	6,595 (34.4)	0.06	2,845 (34.2)	6,528 (34.1)	0.00
Parkinson's	196 (0.7)	75 (0.9)	121 (0.6)	0.19	60 (0.7)	137 (0.7)	0.00
COPD	5,691 (20.7)	1,730 (20.8)	3,961 (20.7)	0.08	1,722 (20.7)	3,970 (20.7)	0.00
Depression	565 (2.1)	189 (2.3)	376 (2.0)	0.07	174 (2.1)	396 (2.1)	0.00
Schizophrenia	(n<10)	(n<10)	(n<10)	.	(n<5)	(n<5)	0.01
Cancer	4,687 (17.1)	1,399 (16.8)	3,288 (17.2)	0.07	1,420 (17.1)	3,269 (17.1)	0.00
**Medications (n,%)**							
Low dose ASA	12,480 (45.4)	3,549 (42.7)	8,931 (46.6)	0.07	3,785 (45.5)	8,708 (45.5)	0.00
ADP inhibitors	297 (1.1)	93 (1.1)	204 (1.1)	0.27	90 (1.1)	208 (1.1)	0.00
Anticoagulants	2,304 (8.4)	583 (7.0)	1,721 (9.0)	0.05	695 (8.4)	1,608 (8.4)	0.00
Thiazides	7,796 (28.4)	2,314 (27.8)	5,482 (28.6)	0.09	2,360 (28.4)	5,437 (28.4)	0.00
Spironolactone	901 (3.3)	256 (3.1)	645 (3.4)	0.13	270 (3.2)	627 (3.3)	0.00
Beta-blockers	7,432 (27.1)	2,063 (24.8)	5,369 (28.0)	0.05	2,244 (27.0)	5,180 (27.0)	0.00
ACE/ARB	16,297 (59.3)	4,651 (56.0)	11,646 (60.8)	0.08	4,936 (59.4)	11,368 (59.4)	0.00
CCBs	10,122 (36.9)	2,849 (34.3)	7,273 (38.0)	0.10	3,070 (36.9)	7,064 (36.9)	0.00
Antidepressants	4,374 (15.9)	1,369 (16.5)	3,005 (15.7)	0.08	1,331 (16.0)	3,058 (16.0)	0.00
Antipsychotics	678 (2.5)	194 (2.3)	484 (2.5)	0.03	207 (2.5)	474 (2.5)	0.00

© 2021 Thompson W et al. *JAMA Network Open.*

NSAIDs (non-selective)	3,789 (13.8)	1,163 (14.0)	2,626 (13.7)	0.06	1,148 (13.8)	2,642 (13.8)	0.00
COX-2 inhibitors	38 (0.1)	10 (0.1)	28 (0.1)	0.20	11 (0.1)	26 (0.1)	0.00
**Cohabitation status (n,%)**							
Alone	12,559 (45.7)	3,603 (43.4)	8,956 (46.8)	.	3,794 (45.7)	8,753 (45.7)	0.00
Cohabiting	14,904 (54.3)	4,708 (56.6)	10,196 (53.2)	.	4,516 (54.3)	10,400 (54.3)	0.00

Abbreviations: ACE = angiotensin converting enzyme; ADP = adenosine diphosphate receptor; ARB = angiotensin receptor blocker; ASA = acetylsalicylic acid; CCB = calcium channel blocker; COPD = chronic obstructive pulmonary disease; COX-2 = cyclooxygenase-2; IQR = interquartile range; NSAID = non-steroidal anti-inflammatory drugs; SMD = standardized mean difference

© 2021 Thompson W et al. *JAMA Network Open.*

eTable 6. Balance of Covariates in Secondary Prevention Cohort.

		Before weighting			After weighting		
	**Overall**	**Discontinuation**	**Continuation**	**SMD**	**Discontinuation**	**Continuation**	**SMD**
	(n=39,955)	(n=9,853)	(n=30,102)		(n=9,855)	(n=30,102)	
**Sex (n,%)**							
Female	18,717 (46.8)	5,261 (53.4)	13,456 (44.7)	0.05	4,606 (46.7)	14,098 (46.8)	0.00
Male	21,238 (53.2)	4,592 (46.6)	16,646 (55.3)	0.01	5,249 (53.3)	16,005 (53.2)	0.00
**Age**							
Median (IQR)	80 (77-84)	81 (78-85)	80 (77-83)	0.19	80 (77-84)	80 (77-84)	0.00
75-84 y (n,%)	31,931 (79.9)	7,372 (74.8)	24,559 (81.6)	0.05	7,837 (79.5)	24,109 (80.1)	0.01
≥85 y (n,%)	8,024 (20.1)	2,481 (25.2)	5,543 (18.4)	0.10	2,018 (20.5)	5,993 (19.9)	0.01
**Statin intensity (n,%)**							
Low/moderate	37,469 (93.8)	9,376 (95.2)	28,093 (93.3)	0.10	9,239 (93.7)	28,229 (93.8)	0.00
High	2,486 (6.2)	477 (4.8)	2,009 (6.7)	0.01	616 (6.3)	1,873 (6.2)	0.00
**Unique medications**							
Median (IQR)	8 (5-11)	8 (5-11)	8 (5-11)	0.04	8 (5-11)	8 (5-11)	0.01
0-4	6,905 (17.3)	1,855 (18.8)	5,050 (16.8)	0.08	1,732 (17.6)	5,162 (17.1)	0.01
5-9	19,605 (49.1)	4,795 (48.7)	14,810 (49.2)	0.09	4,770 (48.4)	14,845 (49.3)	0.02
≥10	13,445 (33.7)	3,203 (32.5)	10,242 (34.0)	0.13	3,354 (34.0)	10,095 (33.5)	0.01
**Comorbidities, n(%)**							
Dementia	4,108 (10.3)	1,313 (13.3)	2,795 (9.3)	0.10	1,022 (10.4)	3,102 (10.3)	0.00
Diabetes	11,702 (29.3)	2,575 (26.1)	9,127 (30.3)	0.09	2,900 (29.4)	8,822 (29.3)	0.00
Atrial fibrillation / flutter	9,079 (22.7)	2,077 (21.1)	7,002 (23.3)	0.09	2,267 (23.0)	6,849 (22.8)	0.01
Heart failure	8,373 (21.0)	1,873 (19.0)	6,500 (21.6)	0.16	2,076 (21.1)	6,312 (21.0)	0.00
Hypertension	21,903 (54.8)	5,344 (54.2)	16,559 (55.0)	0.10	5,401 (54.8)	16,500 (54.8)	0.00
Parkinson's	412 (1.0)	105 (1.1)	307 (1.0)	0.01	104 (1.1)	311 (1.0)	0.00
COPD	11,286 (28.2)	2,708 (27.5)	8,578 (28.5)	0.10	2,800 (28.4)	8,509 (28.3)	0.00
Depression	1,551 (3.9)	405 (4.1)	1,146 (3.8)	0.17	384 (3.9)	1,170 (3.9)	0.00
Schizophrenia	16 (0.0)	(n<10)	(n<10)	0.21	(n<5)	12 (0.0)	0.00
Cancer	7,254 (18.2)	1,738 (17.6)	5,516 (18.3)	0.11	1,783 (18.1)	5,465 (18.2)	0.00
**Medications (n,%)**							
Low dose ASA	30,562 (76.5)	7,294 (74.0)	23,268 (77.3)	0.10	7,532 (76.4)	23,021 (76.5)	0.00
ADP inhibitors	3,263 (8.2)	700 (7.1)	2,563 (8.5)	0.08	812 (8.2)	2,460 (8.2)	0.00
Anticoagulants	5,745 (14.4)	1,241 (12.6)	4,504 (15.0)	0.08	1,436 (14.6)	4,332 (14.4)	0.00
Thiazides	9,160 (22.9)	2,279 (23.1)	6,881 (22.9)	0.10	2,247 (22.8)	6,897 (22.9)	0.00
Spironolactone	2,687 (6.7)	553 (5.6)	2,134 (7.1)	0.15	658 (6.7)	2,024 (6.7)	0.00
Beta-blockers	20,849 (52.2)	4,684 (47.5)	16,165 (53.7)	0.10	5,128 (52.0)	15,701 (52.2)	0.00
ACE/ARB	23,018 (57.6)	5,471 (55.5)	17,547 (58.3)	0.08	5,687 (57.7)	17,341 (57.6)	0.00
CCBs	14,505 (36.3)	3,454 (35.1)	11,051 (36.7)	0.11	3,572 (36.2)	10,930 (36.3)	0.00
Antidepressants	7,958 (19.9)	2,001 (20.3)	5,957 (19.8)	0.14	1,954 (19.8)	5,992 (19.9)	0.00
Antipsychotics	1,007 (2.5)	251 (2.5)	756 (2.5)	0.12	254 (2.6)	761 (2.5)	0.00

© 2021 Thompson W et al. *JAMA Network Open.*

NSAIDs (non-selective)	4,697 (11.8)	1,220 (12.4)	3,477 (11.6)	0.09	1,154 (11.7)	3,538 (11.8)	0.00
COX-2 inhibitors	50 (0.1)	12 (0.1)	38 (0.1)	0.33	12 (0.1)	38 (0.1)	0.00
**Cohabitation status (n,%)**							
Alone	19,571 (49.0)	4,483 (45.5)	15,088 (50.1)	.	4,836 (49.1)	14,748 (49.0)	0.00
Cohabiting	20,384 (51.0)	5,370 (54.5)	15,014 (49.9)	.	5,019 (50.9)	15,355 (51.0)	0.00

Abbreviations: ACE = angiotensin converting enzyme; ADP = adenosine diphosphate receptor; ARB = angiotensin receptor blocker; ASA = acetylsalicylic acid; COPD = chronic obstructive pulmonary disease; COX-2 = cyclooxygenase-2; IQR = interquartile range; NSAID = non-steroidal anti-inflammatory drugs; SMD = standardized mean difference

© 2021 Thompson W et al. *JAMA Network Open.*

eTable 7. Summary of results with grace period of 90 days.

Primary prevention							
	**Discontinuation (11,245 person-years)[a]**		**Continuation (80,686 person-years)[a]**		**Rate difference**		**Subhazard ratio (95% CI)[b]**
**Outcome**	**Crude events**	**Crude incidence rate per 1000 (95% CI)**	**Crude events**	**Crude incidence rate per 1000 (95% CI)**	**Crude rate difference per 1000 (CI)**	**Weighted rate difference per 1000 (CI)[b]**	
**MACE**	357	32 (29 to 35)	1897	24 (22 to 25)	8.2 (4.8 to 11.7)	8.8 (5.3 to 12.3)	1.25 (1.12 to 1.41)
**MI**	102	8.8 (7.3 to 10.7)	507	6.1 (5.6 to 6.7)	2.7 (0.9 to 4.5)	3.0 (1.2 to 4.8)	1.42 (1.14 to 1.77)
**Stroke**	204	18 (16 to 21)	1064	13 (12 to 14)	5.0 (2.4 to 7.6)	5.0 (2.4 to 7.6)	1.21 (1.04 to 1.41)
**Revasc.**	48	4.1 (3.1 to 5.5)	375	4.5 (4.1 to 5.0)	-0.39 (-1.65 to 0.87)	-0.02 (-1.31 to 1.28)	0.96 (0.71 to 1.30)
**Death**	76	6.5 (5.2 to 8.2)	315	3.8 (3.4 to 4.2)	2.8 (1.2 to 4.3)	2.8 (1.3 to 4.4)	1.62 (1.25 to 2.10)
**Secondary prevention**							
	**Discontinuation (12,271 person-years)[a]**		**Continuation (106,010 person-years)[a]**		**Rate difference**		**Subhazard ratio (95% CI)[b]**
**Outcome**	**Crude events**	**Crude incidence rate per 1000 (95% CI)**	**Crude events**	**Crude incidence rate per 1000 (95% CI)**	**Crude rate difference per 1000 (CI)**	**Weighted rate difference per 1000 (CI)[b]**	
**MACE**	761	62 (58 to 67)	5095	48 (47 to 49)	14 (9 to 19)	16(11 to 20)	1.24 (1.15 to 1.35)
**MI**	256	20 (17 to 22)	1820	16 (16 to 17)	3.4 (0.8 to 5.9)	4.5(1.9 to 7.1)	1.21 (1.06 to 1.39)
**Stroke**	360	28 (25 to 31)	2299	21 (20 to 22)	7.3 (4.3 to 10.3)	7.0(4.0 to 10.0)	1.20 (1.07 to 1.35)
**Revasc.**	80	6.1 (4.9 to 7.6)	1125	10 (10 to 11)	-4.0 (-5.5 to -2.6)	-3.4(-4.9 to -1.9)	0.66 (0.53 to 0.84)
**Death**	239	18 (16 to 20)	1074	9.4 (8.9 to 10.0)	8.4 (6.1 to 10.8)	9.0(6.6 to 11.3)	1.76 (1.52 to 2.03)

Abbreviations: CI = confidence interval; Death = death due to myocardial or ischemic stroke; MACE = major adverse cardiovascular events; MI = myocardial infarction; Revasc = revascularization procedure

[a] Person years of follow-up for MACE

[b] Adjusted estimates obtained in an inverse probability of treatment weighted (IPTW) pseudo-population using stabilized weights. Weights incorporated propensity scores calculated from baseline age, sex, number of concomitant medications, individual medications, co-morbidities, statin intensity, and co-habitation status.

© 2021 Thompson W et al. *JAMA Network Open.*

eTable 8. Summary of results with grace period of 30 days.

Primary prevention							
	Discontinuation (7,849 person-years)[a]		Continuation (39,250 person-years)[a]		Rate difference		Subhazard ratio (95% CI)[b]
Outcome	Crude events	Crude incidence rate per 1000 (95% CI)	Crude events	Crude incidence rate per 1000 (95% CI)	Crude rate difference per 1000 (CI)	Weighted rate difference per 1000 (CI)[b]	
**MACE**	245	31 (28 to 35)	883	22 (21 to 24)	8.7 (4.5 to 12.9)	9.4 (5.2 to 13.5)	1.24 (1.07 to 1.44)
**MI**	69	8.6 (6.8 to 10.9)	250	6.2 (5.5 to 7.1)	2.4 (0.2 to 4.5)	2.5 (0.4 to 4.7)	1.23 (0.93 to 1.63)
**Stroke**	135	17 (14 to 20)	460	12 (11 to 13)	5.5 (2.4 to 8.5)	5.6 (2.6 to 8.6)	1.28 (1.05 to 1.56)
**Revasc.**	36	4.5 (3.2 to 6.2)	197	4.9 (4.3 to 5.7)	-0.46 (-2.08 to 1.15)	-0.61 (-2.22 to 0.99)	0.77 (0.53 to 1.11)
**Death**	57	7.1 (5.4 to 9.1)	148	3.7 (3.1 to 4.3)	3.4 (1.5 to 5.3)	3.8 (1.9 to 5.8)	1.98 (1.44 to 2.72)
**Secondary prevention**							
	Discontinuation (8,992 person-years)[a]		Continuation (54,540 person-years)[a]		Rate difference		Subhazard ratio (95% CI)[b]
Outcome	Crude events	Crude incidence rate per 1000 (95% CI)	Crude events	Crude incidence rate per 1000 (95% CI)	Crude rate difference per 1000 (CI)	Weighted rate difference per 1000 (CI)[b]	
**MACE**	568	63 (58 to 69)	2613	48 (46 to 50)	15 (10 to 21)	16(11 to 22)	1.18 (1.07 to 1.29)
**MI**	189	20 (17 to 23)	940	17 (16 to 18)	3.4 (0.4 to 6.5)	4.1(1.0 to 7.1)	1.10 (0.94 to 1.30)
**Stroke**	254	27 (24 to 31)	1103	20 (19 to 21)	7.7 (4.1 to 11.2)	7.1(3.6 to 10.6)	1.14 (0.99 to 1.31)
**Revasc.**	72	7.6 (6.0 to 9.5)	611	11 (10 to 12)	-3.3 (-5.2 to -1.3)	-3.9(-5.8 to -1.9)	0.64 (0.50 to 0.82)
**Death**	179	19 (16 to 21)	542	9.4 (8.7 to 10.3)	9.1 (6.3 to 11.9)	10(8 to 13)	1.83 (1.54 to 2.19)

Abbreviations: CI = confidence interval; Death = death due to myocardial or ischemic stroke; MACE = major adverse cardiovascular events; MI = myocardial infarction; Revasc = revascularization procedure

[a] Person years of follow-up for MACE

[b] Adjusted estimates obtained in an inverse probability of treatment weighted (IPTW) pseudo-population using stabilized weights. Weights incorporated propensity scores calculated from baseline age, sex, number of concomitant medications, individual medications, co-morbidities, statin intensity, and co-habitation status.

© 2021 Thompson W et al. *JAMA Network Open.*

eTable 9. Summary of results with medication possession ratio threshold of ≥ 80%.

Primary prevention							
	Discontinuation (10,395 person-years)[a]		Continuation (96,851 person-years)[a]		Rate difference		Subhazard ratio (95% CI)[b]
Outcome	Crude events	Crude incidence rate per 1000 (95% CI)	Crude events	Crude incidence rate per 1000 (95% CI)	Crude rate difference per 1000 (CI)	Weighted rate difference per 1000 (CI)[b]	
**MACE**	333	32 (29 to 36)	2330	24 (23 to 25)	8.0 (4.4 to 11.6)	8.0(4.3 to 11.6)	1.27 (1.13 to 1.44)
**MI**	86	8.0 (6.5 to 9.9)	646	6.5 (6.0 to 7.0)	1.6 (-0.2 to 3.3)	1.9(0.1 to 3.8)	1.29 (1.02 to 1.63)
**Stroke**	201	19 (17 to 22)	1303	13 (13 to 14)	5.9 (3.2 to 8.7)	5.5(2.7 to 8.2)	1.29 (1.10 to 1.51)
**Revasc.**	37	3.4 (2.5 to 4.7)	456	4.6 (4.2 to 5.0)	-1.1 (-2.3 to 0.0)	-0.24(-1.53 to 1.06)	1.00 (0.71 to 1.41)
**Death**	70	6.5 (5.1 to 8.2)	406	4.0 (3.6 to 4.4)	2.5 (0.9 to 4.0)	2.1(0.5 to 3.6)	1.45 (1.11 to 1.90)
**Secondary prevention**							
	Discontinuation (11,206 person-years)[a]		Continuation (126,948 person-years)[a]		Rate difference		Subhazard ratio (95% CI)[b]
Outcome	Crude events	Crude incidence rate per 1000 (95% CI)	Crude events	Crude incidence rate per 1000 (95% CI)	Crude rate difference per 1000 (CI)	Weighted rate difference per 1000 (CI)[b]	
**MACE**	676	60 (56 to 65)	6180	49 (47 to 50)	12 (7 to 16)	13(8 to 18)	1.29 (1.18 to 1.40)
**MI**	225	19 (17 to 22)	2239	17 (16 to 17)	2.2 (-0.4 to 4.7)	3.5(0.8 to 6.2)	1.24 (1.07 to 1.43)
**Stroke**	357	31 (28 to 34)	2799	21 (20 to 22)	9.4 (6.1 to 12.7)	9.0(5.7 to 12.3)	1.39 (1.24 to 1.56)
**Revasc.**	63	5.2 (4.1 to 6.6)	1345	10 (9 to 11)	-4.8 (-6.2 to -3.4)	-3.7(-5.2 to -2.2)	0.69 (0.53 to 0.90)
**Death**	194	16 (14 to 18)	1332	9.7 (9.2 to 10.2)	6.1 (3.8 to 8.3)	6.2(3.9 to 8.6)	1.57 (1.34 to 1.84)

Abbreviations: CI = confidence interval; Death = death due to myocardial or ischemic stroke; MACE = major adverse cardiovascular events; MI = myocardial infarction; Revasc = revascularization procedure

[a] Person years of follow-up for MACE

[b] Adjusted estimates obtained in an inverse probability of treatment weighted (IPTW) pseudo-population using stabilized weights. Weights incorporated propensity scores calculated from baseline age, sex, number of concomitant medications, individual medications, co-morbidities, statin intensity, and co-habitation status.

© 2021 Thompson W et al. *JAMA Network Open.*

eTable 10. Summary of results with medication possession ratio threshold of ≥ 90%.

Primary prevention							
	**Discontinuation (7,717 person-years)[a]**		**Continuation (78,729 person-years)[a]**		**Rate difference**		**Subhazard ratio (95% CI)[b]**
**Outcome**	**Crude events**	**Crude incidence rate per 1000 (95% CI)**	**Crude events**	**Crude incidence rate per 1000 (95% CI)**	**Crude rate difference per 1000 (CI)**	**Weighted rate difference per 1000 (CI)[b]**	
**MACE**	236	31 (27 to 35)	1876	24 (23 to 25)	6.8 (2.7 to 10.8)	7.1(2.9 to 11.2)	1.21 (1.04 to 1.39)
**MI**	65	8.2 (6.4 to 10.4)	522	6.4 (5.9 to 7.0)	1.8 (-0.3 to 3.8)	2.3(0.1 to 4.5)	1.33 (1.02 to 1.75)
**Stroke**	134	17 (15 to 20)	1036	13 (12 to 14)	4.3 (1.2 to 7.3)	4.1(1.0 to 7.1)	1.16 (0.96 to 1.41)
**Revasc.**	27	3.4 (2.3 to 4.9)	368	4.5 (4.1 to 5.0)	-1.2 (-2.5 to 0.2)	-0.13(-1.65 to 1.39)	1.01 (0.67 to 1.52)
**Death**	56	7.0 (5.4 to 9.1)	337	4.1 (3.7 to 4.6)	2.9 (1.0 to 4.8)	2.4(0.6 to 4.3)	1.49 (1.11 to 2.01)
**Secondary prevention**							
	**Discontinuation (9,026 person-years)[a]**		**Continuation (108,303 person years)[a]**		**Rate difference**		**Subhazard ratio (95% CI)[b]**
**Outcome**	**Crude events**	**Crude incidence rate per 1000 (95% CI)**	**Crude events**	**Crude incidence rate per 1000 (95% CI)**	**Crude rate difference per 1000 (CI)**	**Weighted rate difference per 1000 (CI)[b]**	
**MACE**	528	59 (54 to 64)	5233	48 (47 to 50)	10 (5 to 15)	12(6 to 17)	1.26 (1.15 to 1.39)
**MI**	172	18 (15 to 21)	1887	17 (16 to 17)	1.4 (-1.4 to 4.2)	2.9(-0.0 to 5.9)	1.21 (1.03 to 1.43)
**Stroke**	278	30 (26 to 33)	2366	21 (20 to 22)	8.6 (5.0 to 12.2)	8.3(4.7 to 12.0)	1.37 (1.20 to 1.56)
**Revasc.**	42	4.3 (3.2 to 5.8)	1148	10 (9 to 11)	-5.7 (-7.2 to -4.3)	-4.6(-6.1 to -3.0)	0.60 (0.43 to 0.82)
**Death**	158	16 (14 to 19)	1133	9.7 (9.1 to 10.2)	6.3 (3.7 to 8.8)	6.3(3.7 to 8.9)	1.59 (1.33 to 1.89)

Abbreviations: CI = confidence interval; Death = death due to myocardial or ischemic stroke; MACE = major adverse cardiovascular events; MI = myocardial infarction; Revasc = revascularization procedure

[a] Person years of follow-up for MACE

[b] Adjusted estimates obtained in an inverse probability of treatment weighted (IPTW) pseudo-population using stabilized weights. Weights incorporated propensity scores calculated from baseline age, sex, number of concomitant medications, individual medications, co-morbidities, statin intensity, and co-habitation status.

© 2021 Thompson W et al. *JAMA Network Open.*

eTable 11. Summary of results with no medication possession ratio threshold.

Outcome	Crude events	Crude incidence rate per 1000 (95% CI)	Crude events	Crude incidence rate per 1000 (95% CI)	Crude rate difference per 1000 (CI)	Weighted rate difference per 1000 (CI)[b]	Subhazard ratio (95% CI)[b]
**Primary prevention**							
	**Discontinuation (12,883 person-years)[a]**		**Continuation[a] (107,866 person years)[a]**		**Rate difference**		
**MACE**	419	33 (30 to 36)	2564	24 (23 to 25)	8.8 (5.5 to 12.0)	9.2(5.9 to 12.5)	1.34 (1.20 to 1.49)
**MI**	113	8.5 (7.1 to 10.3)	714	6.4 (6.0 to 6.9)	2.1 (0.5 to 3.8)	2.7(1.0 to 4.4)	1.40 (1.14 to 1.72)
**Stroke**	247	19 (17 to 22)	1440	13 (12 to 14)	5.9 (3.4 to 8.3)	5.4(2.9 to 7.9)	1.31 (1.14 to 1.51)
**Revasc.**	54	4.1 (3.1 to 5.3)	495	4.5 (4.1 to 4.9)	-0.40 (-1.55 to 0.75)	0.51(-0.74 to 1.76)	1.17 (0.88 to 1.56)
**Death**	80	6.0 (4.8 to 7.5)	445	4.0 (3.6 to 4.3)	2.0 (0.7 to 3.4)	2.0(0.6 to 3.3)	1.43 (1.11 to 1.83)
**Secondary prevention**							
	**Discontinuation (13,263 person-years)[a]**		**Continuation (137,508 person-years)[a]**		**Rate difference**		
**Outcome**	**Crude events**	**Crude incidence rate per 1000 (95% CI)**	**Crude events**	**Crude incidence rate per 1000 (95% CI)**	**Crude rate difference per 1000 (CI)**	**Weighted rate difference per 1000 (CI)[b]**	**Subhazard ratio (95% CI)[b]**
**MACE**	801	60 (56 to 65)	6678	49 (47 to 50)	12 (7 to 16)	13(9 to 18)	1.29 (1.19 to 1.40)
**MI**	263	19 (17 to 21)	2405	17 (16 to 17)	2.1 (-0.2 to 4.5)	3.5(1.0 to 5.9)	1.23 (1.08 to 1.41)
**Stroke**	420	30 (28 to 34)	3044	21 (21 to 22)	9.2 (6.2 to 12.2)	8.6(5.5 to 11.6)	1.37 (1.23 to 1.53)
**Revasc.**	81	5.7 (4.6 to 7.0)	1463	10 (10 to 11)	-4.4 (-5.7 to -3.1)	-3.4(-4.8 to -2.0)	0.73 (0.58 to 0.92)
**Death**	222	15 (13 to 17)	1425	9.6 (9.1 to 10.1)	5.7 (3.7 to 7.8)	6.0(3.9 to 8.1)	1.56 (1.34 to 1.80)

Abbreviations: CI = confidence interval; Death = death due to myocardial or ischemic stroke; MACE = major adverse cardiovascular events; MI = myocardial infarction; Revasc = revascularization procedure

[a] Person years of follow-up for MACE

[b] Adjusted estimates obtained in an inverse probability of treatment weighted (IPTW) pseudo-population using stabilized weights. Weights incorporated propensity scores calculated from baseline age, sex, number of concomitant medications, individual medications, co-morbidities, statin intensity, and co-habitation status.

© 2021 Thompson W et al. *JAMA Network Open.*

eTable 12. Summary of results using high dimensional propensity score in inverse probability of treatment weighting procedure.

Outcome	Discontinuation (11,709 person-years)[a] Crude events	Crude incidence rate per 1000 (95% CI)	Continuation (103,664 person years)[a] Crude events	Crude incidence rate per 1000 (95% CI)	Rate difference: Crude rate difference per 1000 (CI)	Weighted rate difference per 1000 (CI)[b]	Subhazard ratio (95% CI)[b]
**Primary prevention**							
**MACE**	382	33 (30 to 36)	2481	24 (23 to 25)	8.7 (5.3 to 12.1)	9.1(5.7 to 12.6)	1.32 (1.17 to 1.49)
**MI**	102	8.5 (7.0 to 10.3)	692	6.5 (6.0 to 7.0)	2.0 (0.3 to 3.7)	2.8(1.0 to 4.6)	1.42 (1.12 to 1.79)
**Stroke**	229	19 (17 to 22)	1390	13 (13 to 14)	6.2 (3.6 to 8.8)	5.4(2.8 to 8.0)	1.29 (1.11 to 1.50)
**Revasc.**	47	3.9 (2.9 to 5.2)	477	4.5 (4.1 to 4.9)	-0.59 (-1.77 to 0.59)	0.45(-0.85 to 1.75)	1.15 (0.82 to 1.60)
**Death**	76	6.3 (5.0 to 7.8)	433	4.0 (3.6 to 4.4)	2.2 (0.8 to 3.7)	2.3(0.8 to 3.7)	1.47 (1.13 to 1.91)
**Secondary prevention**							
	**Discontinuation (12,350 person-years)[a]**		**Continuation (133,374 person-years)[a]**		**Rate difference**		**Subhazard ratio (95% CI)[b]**
**Outcome**	**Crude events**	**Crude incidence rate per 1000 (95% CI)**	**Crude events**	**Crude incidence rate per 1000 (95% CI)**	**Crude rate difference per 1000 (CI)**	**Weighted rate difference per 1000 (CI)[b]**	
**MACE**	739	60 (56 to 64)	6472	49 (47 to 50)	11 (7 to 16)	14(9 to 18)	1.27 (1.17 to 1.38)
**MI**	248	19 (17 to 21)	2326	17 (16 to 17)	2.4 (-0.1 to 4.8)	3.7(1.2 to 6.3)	1.22 (1.06 to 1.41)
**Stroke**	382	30 (27 to 33)	2953	21 (21 to 22)	8.4 (5.4 to 11.5)	8.2(5.0 to 11.3)	1.31 (1.17 to 1.47)
**Revasc.**	74	5.5 (4.4 to 7.0)	1416	10 (10 to 11)	-4.5 (-5.9 to -3.1)	-3.8(-5.2 to -2.4)	0.66 (0.52 to 0.85)
**Death**	211	16 (14 to 18)	1387	9.6 (9.1 to 10.1)	6.0 (3.8 to 8.2)	7.2(4.9 to 9.4)	1.64 (1.40 to 1.92)

Abbreviations: CI = confidence interval; Death = death due to myocardial or ischemic stroke; MACE = major adverse cardiovascular events; MI = myocardial infarction; Revasc = revascularization procedure

[a] Person years of follow-up for MACE

[b] Adjusted estimates obtained in an inverse probability of treatment weighted (IPTW) pseudo-population using stabilized weights. Weights incorporated high dimensional propensity scores calculated from baseline age, sex, statin intensity, co-habitation status, and most common diagnoses and medications.

© 2021 Thompson W et al. *JAMA Network Open.*

eTable 13. Revascularization outcome broken down by PCI and CABG.

Primary prevention							
	Discontinuation (12,126 person years)[a]		Continuation (106,834 person years)[a]		Rate difference		Subhazard ratio (95% CI)[b]
Outcome	Crude events	Crude incidence rate per 1000 (95% CI)	Crude events	Crude incidence rate per 1000 (95% CI)	Crude rate difference per 1000 (CI)	Weighted rate difference per 1000 (CI)[b]	
Revasc.	47	3.9 (2.9 to 5.2)	477	4.5 (4.1 to 4.9)	-0.59 (-1.77 to 0.59)	0.32 (-0.97 to 1.60)	1.12 (0.82 to 1.52)
PCI	41	3.4 (2.5 to 4.6)	345	3.2 (2.9 to 3.6)	0.15 (-0.94 to 1.24)	0.81 (-0.37 to 1.99)	1.25 (0.90 to 1.75)
CABG	6	0.49 (0.22 to 1.10)	133	1.1 (1.1 to 1.5)	-0.75 (-1.20 to -0.30)	-0.50(-1.01 to 0.01)	0.68 (0.30 to 1.55)
**Secondary prevention**							
	Discontinuation (13,336 person years)[a]		Continuation (140,970 person years)[a]		Rate difference		Subhazard ratio (95% CI)[b]
Outcome	Crude events	Crude incidence rate per 1000 (95% CI)	Crude events	Crude incidence rate per 1000 (95% CI)	Crude rate difference per 1000 (CI)	Weighted rate difference per 1000 (CI)[b]	
Revasc.	74	5.5 (4.4 to 7.0)	1416	10 (10 to 11)	-4.5 (-5.9 to -3.1)	-3.4 (-4.8 to -1.9)	0.73 (0.57 to 0.93)
PCI	69	5.2 (4.1 to 6.6)	1206	8.6 (8.1 to 9.1)	-3.4 (-4.7 to -2.1)	-2.2(-3.7 to -0.9)	0.79 (0.61 to 1.01)
CABG	5	0.37 (0.16 to 0.90)	214	1.5 (1.3 to 1.7)	-1.1 (-1.5 to -0.8)	-1.1(-1.5 to -0.7)	0.32 (0.13 to 0.77)

Abbreviations: CABG = coronary artery bypass graft; CI = confidence interval; PCI = percutaneous coronary intervention; Revasc = revascularization procedure

[a] Person years of follow-up for revascularizations

[b] Adjusted estimates obtained in an inverse probability of treatment weighted (IPTW) pseudo-population using stabilized weights. Weights incorporated propensity scores calculated from baseline age, sex, number of concomitant medications, individual medications, co-morbidities, statin intensity, and co-habitation status. The grace period (180 days) and medication possession ratio cutoff (70%) were the same as for the main analysis.

© 2021 Thompson W et al. *JAMA Network Open.*

eTable 14. General Practitioner (GP) contacts[a] each year of follow-up.

	Incidence of GP contacts per 1000 persons	
**Year**	**Discontinuation**	**Continuation**
Primary prevention		
2011[b]	2912	6414
2012	6380	11750
2013	7953	11593
2014	9471	11594
2015	10138	11754
2016	10084	11560
Secondary prevention		
2011[b]	3411	7242
2012	7144	13123
2013	8833	12892
2014	10296	12991
2015	11010	12999
2016	11073	12665

[a] Included any GP contact (e.g. in-person visit, home visit, telephone consultation, email)
[b] Follow up started June 30, 2011

© 2021 Thompson W et al. *JAMA Network Open.*

eTable 15. Crude incidence of mortality for causes other than MI and ischemic stroke.

	**Incidence of mortality per 1000**	
**Year of follow-up**	**Discontinuation**	**Continuation**
Primary		
1	130 (105-161)	42 (39-44)
2	147 (129-167)	49 (46-52)
3	113 (100-128)	46 (43-49)
4	125 (112-139)	53 (50-57)
5	148 (135-163)	57 (54-61)
>5	176 (157-198)	60 (54-67)
**Overall**	139 (132-145)	49 (48-50)
Secondary		
1	209 (179-243)	42 (39-44)
2	211 (191-233)	49 (46-52)
3	192 (176-210)	46 (43-49)
4	209 (193-227)	53 (50-57)
5	231 (214-249)	57 (54-61)
>5	247 (223-274)	60 (54-67)
**Overall**	216 (208-224)	49 (48-50)

© 2021 Thompson W et al. *JAMA Network Open.*

eTable 16. Other cardiovascular medication use approaching statin discontinuation date[a] in statin discontinuation group.

Medication	180 day period before discontinuation date	181 to 360 day period before discontinuation	Baseline
Primary			
ASA	2651 (32%)	3213 (39%)	3549 (43%)
Thiazides	1631 (20%)	1940 (23%)	2314 (28%)
ACE/ARB	3954 (48%)	4531 (55%)	4651 (56%)
Secondary			
ASA	5314 (54%)	6398 (65%)	7294 (74%)
Thiazides	1562 (16%)	1920 (19%)	2279 (23%)
ACE/ARB	4397 (45%)	5174 (53%)	5471 (56%)

Abbreviations: ACE/ARB = angiotensin converting enzyme inhibitor or angiotensin receptor blocker; ASA = aspirin

[a] end of grace period

© 2021 Thompson W et al. *JAMA Network Open.*

eTable 17. Crude incidence of MACE each year of follow-up.

	Incidence of MACE per 1000 persons	
**Year of follow-up**	**Discontinuation**	**Continuation**
Primary		
1	36 (24-54)	24 (22-26)
2	33 (25-43)	22 (20-24)
3	37 (30-46)	25 (22-27)
4	32 (26-39)	25 (22-27)
5	31 (25-38)	26 (23-29)
>5	29 (22-39)	21 (18-25)
Secondary		
1	75 (58-97)	53 (51-55)
2	60 (50-72)	51 (49-54)
3	62 (53-73)	45 (43-48)
4	59 (50-68)	48 (45-51)
5	57 (49-66)	43 (40-47)
>5	56 (45-69)	39 (35-44)

© 2021 Thompson W et al. *JAMA Network Open.*

eTable 18. Summary of results for all-cause and non-cardiovascular mortality .

Primary prevention							
	Discontinuation (12,239 person years)[a]		Continuation (108,930 person years)[a]		Rate difference		Subhazard ratio (95% CI)[b]
Outcome	Crude events	Crude incidence rate per 1000 (95% CI)	Crude events	Crude incidence rate per 1000 (95% CI)	Crude rate difference per 1000 (CI)	Weighted rate difference per 1000 (CI)[b]	
All-cause	1873	153 (146 to 160)	6158	57 (55 to 58)	97 (89 to 104)	89 (82 to 96)	2.35 (2.22 to 2.49)
Non-CV	1775	146 (139 to 153)	5642	52 (51 to 54)	94 (87 to 101)	87(80 to 93)	2.42 (2.28 to 2.56)
**Secondary prevention**							
	Discontinuation (13,768 person years)[a]		Continuation (147,668 person years)[a]		Rate difference		Subhazard ratio (95% CI)[b]
Outcome	Crude events	Crude incidence rate per 1000 (95% CI)	Crude events	Crude incidence rate per 1000 (95% CI)	Crude rate difference per 1000 (CI)	Weighted rate difference per 1000 (CI)[b]	
All-cause	3369	245 (237 to 253)	14527	98 (97 to 100)	146 (138 to 155)	140(131 to 148)	2.29 (2.20 to 2.39)
Non-CV	3073	227 (219 to 235)	12664	88 (86 to 89)	139 (131 to 148)	133(124 to 141)	2.38 (2.28 to 2.48)

Abbreviations: CI = confidence interval; CV = cardiovascular

[a] Person years of follow-up for all-cause mortality

[b] Adjusted estimates obtained in an inverse probability of treatment weighted (IPTW) pseudo-population using stabilized weights. Weights incorporated propensity scores calculated from baseline age, sex, number of concomitant medications, individual medications, co-morbidities, statin intensity, and co-habitation status. The grace period (180 days) and medication possession ratio cutoff (70%) were the same as for the main analysis.

© 2021 Thompson W et al. *JAMA Network Open.*

eTable 19. Summary of results for negative control outcome (hip fracture).

Primary prevention							
	**Discontinuation (10,884 person years)[a]**		**Continuation (102,406 person years)[a]**		**Rate difference**		**Subhazard ratio (95% CI)[b]**
**Outcome**	**Crude events**	**Crude incidence rate per 1000 (95% CI)**	**Crude events**	**Crude incidence rate per 1000 (95% CI)**	**Crude rate difference per 1000 (CI)**	**Weighted rate difference per 1000 (CI)[b]**	
Hip fract[c]	225	21 (18 to 24)	1169	11 (11 to 12)	9.3 (6.5 to 12.0)	7.6(4.8 to 10.3)	1.51 (1.30 to 1.75)
**Secondary prevention**							
	**Discontinuation (12,011 person years)[a]**		**Continuation (137,361 person years)[a]**		**Rate difference**		**Subhazard ratio (95% CI)[b]**
**Outcome**	**Crude events**	**Crude incidence rate per 1000 (95% CI)**	**Crude events**	**Crude incidence rate per 1000 (95% CI)**	**Crude rate difference per 1000 (CI)**	**Weighted rate difference per 1000 (CI)[b]**	
Hip fract[c]	308	26 (23 to 29)	2017	15 (14 to 15)	11 (8 to 14)	8.5(5.6 to 11.4)	1.45 (1.28 to 1.64)

Abbreviations: fract = fracture

[a] Person years of follow-up for hip fracture

[b] Adjusted estimates obtained in an inverse probability of treatment weighted (IPTW) pseudo-population using stabilized weights. Weights incorporated propensity scores calculated from baseline age, sex, number of concomitant medications, individual medications, co-morbidities, statin intensity, and co-habitation status. The grace period (180 days) and medication possession ratio cutoff (70%) were the same as for the main analysis.

[c] Hip fracture was determined using the following ICD-10 codes: DS720, DS721, DS722 (Hjeholt et al. Clinical Epidemiology 2020:12 123–131)

© 2021 Thompson W et al. *JAMA Network Open.*

eTable 20. Summary of results sex-stratified MACE analysis.

Primary prevention							
	Discontinuation (3,048-m; 8,661-f)[a]		Continuation (34,470-m; 68,925-f)[a]		Rate difference		Subhazard ratio (95% CI)[b]
Outcome	Crude events	Crude incidence rate per 1000 (95% CI)	Crude events	Crude incidence rate per 1000 (95% CI)	Crude rate difference per 1000 (CI)	Weighted rate difference per 1000 (CI)[b]	
MACE-m	127	42 (35 to 50)	1041	30 (28 to 32)	12 (4 to 19)	12(5 to 19)	1.34 (1.10 to 1.63)
MACE-f	255	29 (26 to 33)	1440	21 (20 to 22)	8.5 (4.8 to 12.3)	7.7(3.8 to 11.6)	1.32 (1.15 to 1.52)
**Secondary prevention**							
	Discontinuation (5,197-m; 7,153-f)[a]		Continuation (70,192-m; 63,182-f)[a]		Rate difference		Subhazard ratio (95% CI)[b]
Outcome	Crude events	Crude incidence rate per 1000 (95% CI)	Crude events	Crude incidence rate per 1000 (95% CI)	Crude rate difference per 1000 (CI)	Weighted rate difference per 1000 (CI)[b]	
MACE-m	327	63 (56 to 70)	3734	53 (52 to 55)	9.7 (2.7 to 16.8)	12(6 to 19)	1.21 (1.08 to 1.36)
MACE-f	412	58 (52 to 63)	2738	43 (42 to 45)	14 (8 to 20)	14(7 to 20)	1.36 (1.22 to 1.52)

Abbreviations: f = female; m = male; MACE-m = major adverse cardiovascular events in males to MACE-w = major adverse cardiovascular events in females

[a] Person years of follow-up for MACE

[b] Adjusted estimates obtained in an inverse probability of treatment weighted (IPTW) pseudo-population using stabilized weights. Weights incorporated propensity scores calculated from baseline age, number of concomitant medications, individual medications, co-morbidities, statin intensity, and co-habitation status. The grace period (180 days) and medication possession ratio cutoff (70%) were the same as for the main analysis.

© 2021 Thompson W et al. *JAMA Network Open.*

STROBE Statement—Checklist of items that should be included in reports of cohort studies

	Item No	Recommendation	Page No
**Title and abstract**	1	(*a*) Indicate the study's design with a commonly used term in the title or the abstract	
		(*b*) Provide in the abstract an informative and balanced summary of what was done and what was found	3
**Introduction**			
Background/rationale	2	Explain the scientific background and rationale for the investigation being reported	4
Objectives	3	State specific objectives, including any prespecified hypotheses	4
**Methods**			
Study design	4	Present key elements of study design early in the paper	5
Setting	5	Describe the setting, locations, and relevant dates, including periods of recruitment, exposure, follow-up, and data collection	5,6
Participants	6	(*a*) Give the eligibility criteria, and the sources and methods of selection of participants. Describe methods of follow-up	5,6
		(*b*) For matched studies, give matching criteria and number of exposed and unexposed	
Variables	7	Clearly define all outcomes, exposures, predictors, potential confounders, and effect modifiers. Give diagnostic criteria, if applicable	6, supp material
Data sources/ measurement	8*	For each variable of interest, give sources of data and details of methods of assessment (measurement). Describe comparability of assessment methods if there is more than one group	5, 6, supp material
Bias	9	Describe any efforts to address potential sources of bias	7
Study size	10	Explain how the study size was arrived at	5
Quantitative variables	11	Explain how quantitative variables were handled in the analyses. If applicable, describe which groupings were chosen and why	7
Statistical methods	12	(*a*) Describe all statistical methods, including those used to control for confounding	7
		(*b*) Describe any methods used to examine subgroups and interactions	
		(*c*) Explain how missing data were addressed	
		(*d*) If applicable, explain how loss to follow-up was addressed	
		(*e*) Describe any sensitivity analyses	
**Results**			

© 2021 Thompson W et al. *JAMA Network Open.*

Participants	13*	(a) Report numbers of individuals at each stage of study—eg numbers potentially eligible, examined for eligibility, confirmed eligible, included in the study, completing follow-up, and analysed	9
		(b) Give reasons for non-participation at each stage	
		(c) Consider use of a flow diagram	
Descriptive data	14*	(a) Give characteristics of study participants (eg demographic, clinical, social) and information on exposures and potential confounders	9, Table 1 and 2
		(b) Indicate number of participants with missing data for each variable of interest	
		(c) Summarise follow-up time (eg, average and total amount)	
Outcome data	15*	Report numbers of outcome events or summary measures over time	9, 10

© 2021 Thompson W et al. *JAMA Network Open.*

Main results	16	(*a*) Give unadjusted estimates and, if applicable, confounder-adjusted estimates and their precision (eg, 95% confidence interval). Make clear which confounders were adjusted for and why they were included	9, 10
		(*b*) Report category boundaries when continuous variables were categorized	
		(*c*) If relevant, consider translating estimates of relative risk into absolute risk for a meaningful time period	
Other analyses	17	Report other analyses done—eg analyses of subgroups and interactions, and sensitivity analyses	9, 10
**Discussion**			
Key results	18	Summarise key results with reference to study objectives	12
Limitations	19	Discuss limitations of the study, taking into account sources of potential bias or imprecision. Discuss both direction and magnitude of any potential bias	13,14
Interpretation	20	Give a cautious overall interpretation of results considering objectives, limitations, multiplicity of analyses, results from similar studies, and other relevant evidence	14
Generalisability	21	Discuss the generalisability (external validity) of the study results	13,14
**Other information**			
Funding	22	Give the source of funding and the role of the funders for the present study and, if applicable, for the original study on which the present article is based	17

*Give information separately for exposed and unexposed groups.

Note: An Explanation and Elaboration article discusses each checklist item and gives methodological background and published examples of transparent reporting. The STROBE checklist is best used in conjunction with this article (freely available on the Web sites of PLoS Medicine at http://www.plosmedicine.org/, Annals of Internal Medicine at http://www.annals.org/, and Epidemiology at http://www.epidem.com/). Information on the STROBE Initiative is available at http://www.strobe-statement.org.
